# Spatial differentiation of metabolism in prostate cancer tissue by MALDI-TOF MSI

**DOI:** 10.1186/s40170-021-00242-z

**Published:** 2021-01-29

**Authors:** Maria K. Andersen, Therese S. Høiem, Britt S. R. Claes, Benjamin Balluff, Marta Martin-Lorenzo, Elin Richardsen, Sebastian Krossa, Helena Bertilsson, Ron M. A. Heeren, Morten B. Rye, Guro F. Giskeødegård, Tone F. Bathen, May-Britt Tessem

**Affiliations:** 1 Department of Circulation and Medical Imaging, NTNU–Norwegian University of Science and Technology, Trondheim, Norway; 2 Maastricht MultiModal Molecular Imaging institute (M4I), Maastricht University, Maastricht, The Netherlands; 3 Department of Medical Biology, UiT The Artic University of Norway, Tromsø, Norway; 4 Department of Clinical Pathology, University Hospital of North Norway, UNN, Tromsø, Norway; 5 Department of Clinical and Molecular Medicine, NTNU–Norwegian University of Science and Technology, Trondheim, Norway; 6 Department of Urology, St. Olavs Hospital, Trondheim University Hospital, Trondheim, Norway; 7 Clinic of Surgery, St. Olavs Hospital, Trondheim University Hospital, Trondheim, Norway; 8 Clinic of Laboratory Medicine, St. Olavs Hospital, Trondheim University Hospital, Trondheim, Norway; 9 BioCore-Bioinformatics Core Facility, NTNU–Norwegian University of Science and Technology, Trondheim, Norway

**Keywords:** Prostate cancer, Metabolism, Mass spectrometry imaging, Tumor heterogeneity

## Abstract

**Background:**

Prostate cancer tissues are inherently heterogeneous, which presents a challenge for metabolic profiling using traditional bulk analysis methods that produce an averaged profile. The aim of this study was therefore to spatially detect metabolites and lipids on prostate tissue sections by using mass spectrometry imaging (MSI), a method that facilitates molecular imaging of heterogeneous tissue sections, which can subsequently be related to the histology of the same section.

**Methods:**

Here, we simultaneously obtained metabolic and lipidomic profiles in different prostate tissue types using matrix-assisted laser desorption/ionization time-of-flight (MALDI-TOF) MSI. Both positive and negative ion mode were applied to analyze consecutive sections from 45 fresh-frozen human prostate tissue samples (*N* = 15 patients). Mass identification was performed with tandem MS.

**Results:**

Pairwise comparisons of cancer, non-cancer epithelium, and stroma revealed several metabolic differences between the tissue types. We detected increased levels of metabolites crucial for lipid metabolism in cancer, including metabolites involved in the carnitine shuttle, which facilitates fatty acid oxidation, and building blocks needed for lipid synthesis. Metabolites associated with healthy prostate functions, including citrate, aspartate, zinc, and spermine had lower levels in cancer compared to non-cancer epithelium. Profiling of stroma revealed higher levels of important energy metabolites, such as ADP, ATP, and glucose, and higher levels of the antioxidant taurine compared to cancer and non-cancer epithelium.

**Conclusions:**

This study shows that specific tissue compartments within prostate cancer samples have distinct metabolic profiles and pinpoint the advantage of methodology providing spatial information compared to bulk analysis. We identified several differential metabolites and lipids that have potential to be developed further as diagnostic and prognostic biomarkers for prostate cancer. Spatial and rapid detection of cancer-related analytes showcases MALDI-TOF MSI as a promising and innovative diagnostic tool for the clinic.

**Supplementary Information:**

The online version contains supplementary material available at 10.1186/s40170-021-00242-z.

## Introduction

Molecular characterization of prostate cancer tissue is important in the quest of finding new biomarkers, validate previously suggested clinical biomarkers and to identify potential treatment targets. However, prostate tissue samples are highly heterogeneously composed, containing a mix of normal epithelium, hyperplasia, stroma, and cancer. Due to inherent functional and molecular differences between these tissue components, methodological approaches based on bulk measurements result in average measurements and lost information from the different tissue types. Stromal content in particular can be a confounder in bulk measurements due to the marked difference in the morphological and functional roles of epithelial and stromal cells, where the epithelial cells are responsible for producing the prostatic fluid and stroma offers physical support and contraction of the gland [1]. Understanding the molecular processes specific to each tissue type may provide novel and clinically important insights into the molecular mechanisms of prostate cancer compared to healthy prostate epithelium. Mass spectrometry imaging (MSI) allows for spatial detection of several different classes of potential cancer markers on tissue sections, including metabolites [2, 3], lipids [4, 5], peptides [6], glycans [7], and metals [8], which can be matched to corresponding histology images.

Altered metabolism is a hallmark of cancer [9] and several metabolites and metabolic pathways are differently expressed in prostate cancer [10]. Molecular components associated with healthy prostatic function, such as the metabolites spermine and citrate, and the metal zinc, have long been known to have reduced levels during cancer progression [11, 12], and to be associated with a worse clinical outcome [13, 14]. Lower levels of citrate in prostate cancer compared to normal tissue have also been observed with MSI using desorption electrospray ionization (DESI) [15, 16]. We have recently demonstrated reduced levels of citrate and aspartate, along with the metal zinc, in prostate cancer tissue using matrix-assisted laser desorption ionization (MALDI) MSI [17]. Lipid metabolism is another key metabolic alteration in prostate cancer, including both increased fatty acid synthesis (FAS) and energy utilization of lipids through β-oxidation. Components of the carnitine shuttle, a system to transport fatty acids into the mitochondria for β-oxidation, are reported to have higher levels in cancer compared to normal tissue [18], which is also shown by MSI [19, 20]. FAS is needed for membrane production for cell growth, and increased levels of building blocks required for phospholipid synthesis, such as choline, phosphocholine, glycerophosphocholine, and phosphoethanolamine are reported in prostate cancer [12, 21, 22]. Phospholipid mapping by MSI is particularly beneficial due to their high metabolic stability, amphiphilic chemistry, and confinement to membranes, making them robust against diffusion. Several studies using MSI have demonstrated alterations in phospholipid composition in prostate cancer compared to healthy prostate tissue [3, 4, 15, 16, 19, 23].

The aim of the presented study was to investigate and compare the metabolic and lipidomic profile of different tissue types, including non-cancer epithelium (NCE), stroma, and cancer of prostate tissue samples using MALDI-TOF MSI. Using both negative and positive ion detection mode on serial tissue sections allowed us to simultaneously analyze a range of different metabolites and lipids for the same samples.

## Materials and methods

### Patient inclusion and sample collection

This study was approved by the regional ethical committee of Central Norway (identifier 2017/576), and all methods were performed according to national and EU ethical regulations, as well as the principles of the Declaration of Helsinki. All patients gave a written informed consent before tissue specimens were collected.

Specimens were collected from 15 prostate cancer patients undergoing radical prostatectomy at St. Olav’s University Hospital in the period 2007–2008. The patients had a mean age of 63.7 (range 48−69) at the time of surgery, median post-operative T-stage T2c (range T2c−T3b), median post-operative Grade Group 3 (range 2−5) and mean pre-operative serum PSA of 11.09 ng/mL (range 5.2−21.4). Further clinical details can be found in Supplementary Table S1. A 2-mm-thick tissue slice was removed from the middle of the prostate, snap frozen, and stored at – 80 °C as described by Bertilsson et al. [24]. A range of 1 to 6 fresh frozen tissue core samples (3 mm in diameter) were drilled from each slice, giving a total of 45 samples. The samples included in this study were originally collected as part of a previous sample cohort [12, 13, 21, 24, 25], but were not used for analysis at that time.

### Sample preparation

The tissue samples were cryosectioned with 4 μm thickness and sections were thaw-mounted onto indium tin oxide (ITO) coated glass slides (Bruker Daltonics, part nr. 9237001, Bremen, Germany). Two sections were cut from each sample to give two sets, one for positive and one for negative ion mode (*n* = 90 sections in total), and four to five sections were placed on each ITO-slide in a randomized order and were stored at – 80 °C until further use. Performing analysis in both ion modes allowed us to detect a wider number of molecules. This is related to the different chemical properties of biological analytes, which leaves certain masses to be exclusively detected with MS in either positive or negative ion mode.

All ITO-slides with tissue sections were moved directly from – 80 °C storage to a vacuum chamber and dried for a minimum of 20 min prior to matrix application. Two different matrixes, 2,5-dihydroxybenzoic acid (DHB) and *N*-(1-naphthyl) ethylenediamine dihydrochloride (NEDC) were prepared; DHB (20 mg/ml) was dissolved in 70% methanol/0.1% trifluoroacetic acid and NEDC (7 mg/ml) was dissolved in 70% methanol. Matrix was sprayed onto the tissue sections with the HTX TM-Sprayer^TM^ system (HTX Technology), with 10 and 14 layers of matrix for DHB and NEDC, respectively. Details of spraying parameters can be found in Supplementary Table S2. All tissue sections coated with the same matrix were prepared and measured with MALDI-TOF MSI on the same day to minimize day-to-day variation.

### MALDI-TOF measurement

All tissue sections were measured on a rapifleX^TM^ MALDI Tissuetyper^TM^ (Bruker Daltonics) equipped with a 10 kHz laser shooting 200 shots per pixel at a 10 kHz frequency with a spatial resolution of 30 μm. Red phosphorus was used to calibrate the instrument prior to all measurements. The tissue sections covered with DHB matrix were measured in positive ion mode with a mass range of *m/z* 100–1000, while tissue sections covered with NEDC matrix were measured in the mass range *m/z* 40–1000 in negative ion mode. Separate matrix-only regions were recorded for all measurements. After data acquisition, the slides were stored at 4 °C until staining with hematoxylin and eosin (H&E).

### Data preprocessing and peak selection

Due to spectral shifts between all imaging experiments in positive ion mode, a ‘Cubic Enhanced’ recalibration (also termed peak-realignment) was performed in FlexAnalysis (Bruker Daltonics) to align spectra to each other. Seven masses were used to calibrate, of which four were matrix-related (*m/z* 155.08, 273.08, 348.23, and 439.12) and present on all spectra, while three were present on tissue spectra (*m/z* 104.17–choline, *m/z* 496.12–unidentified, and *m/z* 782.65–phosphatidylcholine 16:0_18:1). A maximum tolerance of 400 ppm was used for *m/z* 104.17 and 200 ppm was used for the other calibrant masses.

Stained H&E images were co-registered with the MALDI MSI data in FlexImaging v4.1 (Bruker Daltonics). Histopathology was performed by a trained uropathologist (E.R.) resulting in digital annotations for stroma (including stroma from benign areas and tumor), NCE (including normal glands and hyperplasia), and cancer.

Measurements with the same matrix and ion mode were merged into one dataset in the MSI data analysis environment SCiLS Lab 2020a (Bruker Daltonics), where each spectrum was normalized on its total ion count. Global, cross-patient mean spectra were calculated for stroma, NCE, cancer, and matrix-only spectra separately and uploaded into mMass v.5.5.0 [26] for peak detection. In mMass, all mean spectra were baseline corrected (precision = 20, relative offset = 25) before the mean matrix spectrum was subtracted from the other NCE, cancer, and stroma mean spectra. Peak selection was then performed on the resulting mean-spectra (with matrix peaks removed) with a minimum S/N of 2, deisotoping and an absolute intensity threshold of 0.2 and 0.25 for negative and positive ion mode spectra, respectively. Isotopes not automatically removed by mMass were removed through manual assessment. In addition, lipid fragments identified from the MS/MS spectra were also removed from the mass list. The peaks selected from the mean spectra of stroma, NCE, and cancer were combined and duplicate peaks were removed, resulting in two final peak lists of 167 and 136 for the positive and negative ion mode datasets, respectively.

All spectra were exported from SCiLS Lab and an in-house build script in R was used to reduce each spectrum by selecting the highest data point within each peak interval. Due to minor mass shifts from spectrum to spectrum, the intervals were 200 and 150 ppm for negative and positive ion mode spectra, respectively, and were used to locate the peaks and their highest data point.

### Data analysis and statistics

The reduced data was imported into MATLAB v.9.3 (MathWorks, Natick, USA), and multivariate analysis was performed on auto scaled data using PLS_Toolbox v.8.6.2 (Eigenvector Research Inc., Manson, USA). Unsupervised principal component analysis (PCA) was applied to investigate the presence of any natural clustering based on histology type and/or patient origin. Two PCA models were created, one for each ion mode, which were necessary as the data sets were acquired from separate tissue sections which could not be exactly overlaid. Further, the supervised multivariate method Orthogonal Partial Least Squares Discriminant Analysis (OPLS-DA) was applied to pairwise compare NCE, stroma, and cancer, for both ion mode datasets separately, producing a total of six different models. The leave-one-patient-out approach was used for cross-validation, meaning that all spectra originating from the same patient were left out during a cross-validation cycle. Permutation testing (1000 iterations) was performed to test significance, defined as *p* ≤ 0.05, of all six OPLS-DA models. Variable importance on the projection (VIP) score was used to identify the main differing masses between the tissue types. The VIP score indicates the importance of specific variables (in this case masses) when creating a supervised PLS model, and variables with VIP ≥ 1 are generally considered important for discrimination [27].

Univariate linear mixed models (LMM) were also applied to the reduced datasets with extracted masses in R using the *nlme* v3.1-137 package [28]. We used a fraction-based iterative adaption of LMM to account for spatial autocorrelation common to MSI [29]. With this approach, 0.5% of all spectra are randomly selected and used for LMM to minimize the number of neighboring spectra being analyzed in the same model. This step was repeated 1000 times and the reported Benjamini-Hochberg adjusted *p* value (< 0.05 were considered significant) is the average *p* value from all 1000 iterations. Further details and the full R script is provided in a previous publication [17]. Additionally, log_2_ fold changes (log_2_FC) were calculated to show the differences in levels between the tissue types for all extracted masses.

### Identification of masses

Separate tissue sections, originating from samples that were equivalent (same collection method, storage time and sectioning conditions) to those used for the MALDI-TOF MSI, were prepared and sprayed with the same protocol as described above. Based on previous biological knowledge, visual mass distributions and VIP scores from the OPLS-DA models, a subset of masses were selected for identification through tandem mass spectrometry (MS/MS). MS/MS were performed on a high mass resolution Q Exactive HF Hybrid Quadrupole-Orbitrap (Thermo Fisher Scientific GmbH, Bremen, Germany) coupled to a MALDI-ESI injector (Spectroglyph, LLC, Kennewick, WA, USA). MS/MS spectra were acquired in both polarities using a high-energy collisional dissociation cell with a ± 0.5 Da isolation window, normalized collision energy (range 10–80, manufacturer units), 1000 Hz laser frequency, and a mass resolution of 240,000 (FWHM at *m/z* 200). For each precursor mass, 20 spectra were acquired for with an injection time of 2000 ms per scan while continuously moving the MALDI stage. Due to lower intensities of the nucleotides AMP, ADP, and ATP, these masses were identified through MS/MS acquisition on a tims-TOF flex (Bruker Daltonics) using MALDI-2, an isolation window of 1 Da and 25 eV collision energy. A total of 175, 125, and 250 laser shots were fired at a 1 kHz frequency for AMP, ADP, and ATP, respectively. All metabolites and lipids were identified by accurate mass from Orbitrap acquisition and by manually assessing the fragment pattern and comparing the averaged MS/MS spectra to those in the data bases METLIN [30], the Human Metabolome Database (HMDB) [31], and Alex^123^ Lipid Calculator [32]. The metal zinc (in form of ZnCl_3_^-^) was identified through isotopic peak pattern, accurate mass, and laser ablation inductively coupled plasma (LA-ICP) MSI as described in our previous publication [17].

## Results

### Sample and analysis overview

MALDI-TOF MS imaging of 45 fresh frozen prostate tissue sections resulted in a total of ~ 188,000 spectra for both negative and positive ion mode with ~ 83,000 spectra from stroma, ~ 65,000 spectra from cancer, and ~ 40,000 spectra for NCE (Supplementary Table S3). After removal of matrix and isotopic signals, 167 and 136 detected peaks remained in the mean spectra for positive and negative ion mode measurements, respectively. Of the selected peaks, a total of 27 metabolites and 44 lipids were successfully identified for both ion modes (Supplementary Table S4). The mean peak height for each mass in stroma, NCE, and cancer tissue are presented in Supplementary Table S5 and S6.

**Fig. 1:**
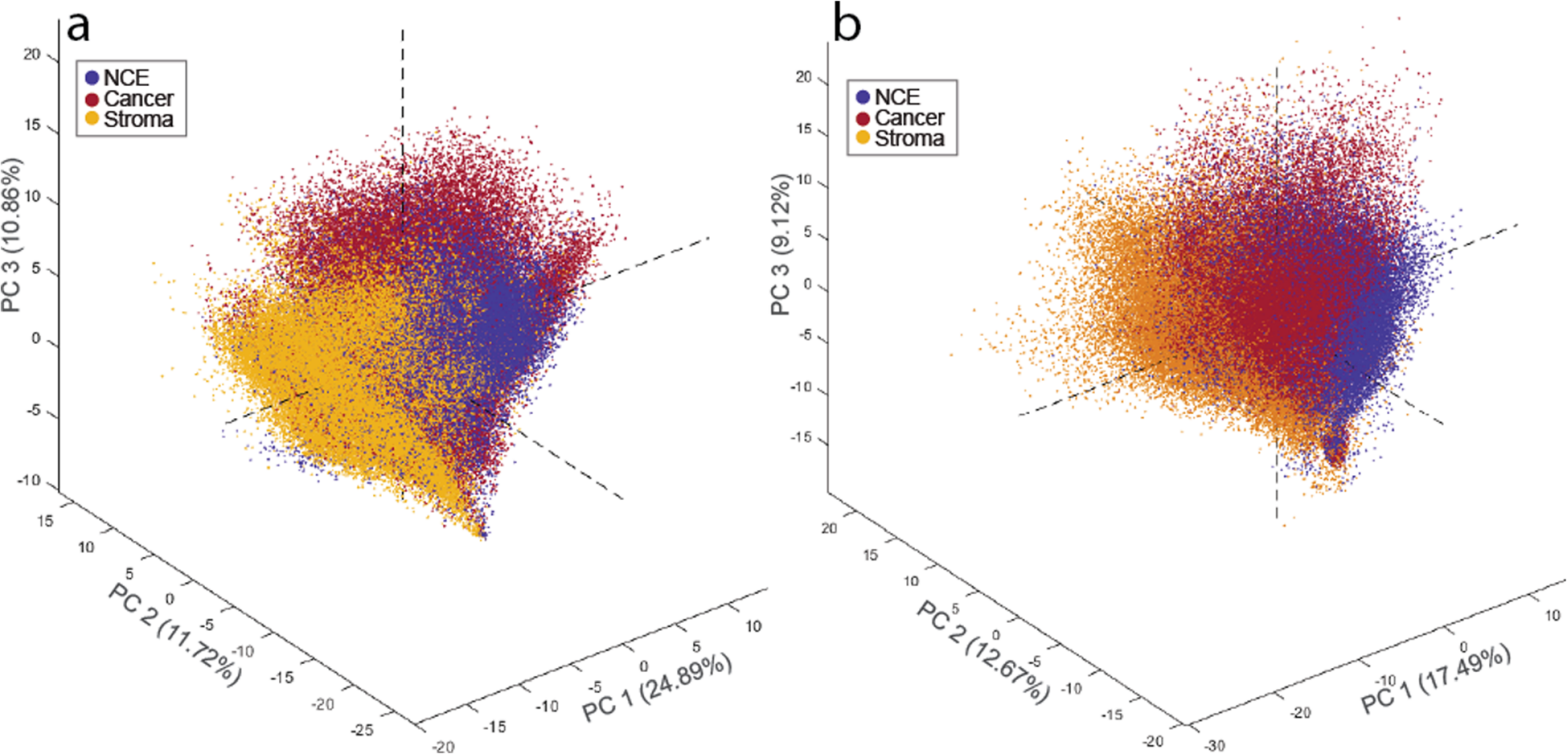
Scores plot from principal component analysis (PCA) of metabolites and lipids detected with MALDI MSI. The three first principal components (PC) are shown for both **a** negative and **b** positive ion mode data sets. Data points represent single mass spectra and are colored by tissue type: non-cancer epithelium (NCE, blue), cancer (red), and yellow (stroma)

**Fig. 2:**
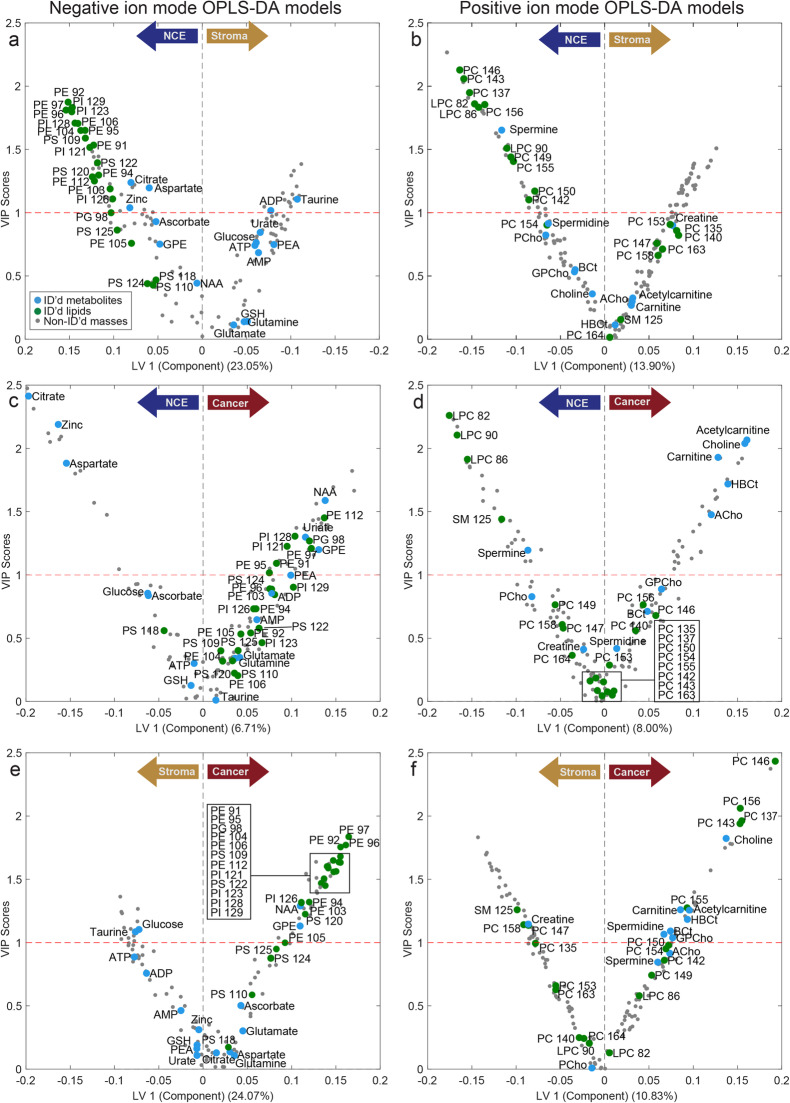
Metabolic differences between non-cancer epithelium (NCE), stroma, and cancer identified by orthogonalized partial least squares discrimination analysis (OPLS-DA). The first OPLS-DA latent variable is the main separator between the different tissue types (indicated by left and right arrows) and are represented on the *x*-axis, while the *y*-axis represents the variable importance on the projection (VIP) score for each mass. VIP > 1 for a variable is considered an important influence on the model and is indicated as a red dotted line. For visual purposes, the loading scores in **a** have been reversed. Identified metabolites and lipids are shown as blue and green circles, respectively. ACho = acetylcholine, ADP = adenosine diphosphate, AMP = adenosine monophosphate, ATP = adenosine triphosphate, BCt = butyryl-L-carnitine, GPE = glycerophoshpoethanolamine, HBCt = hydroxybutyrylcarnitine, NAA = *N*-acetylaspartate, LPC = lysophosphatidylcholines, PCho = phosphocholine, PE = phosphoethanolamines, PE = phosphatidylethanolamines, PC = phosphatidylcholines, PG = phosphatidylglycerols, PI = phosphatidylinositols, PS = phosphatidylserine, and SM = sphingomyelin. The particular phospholipids and their chain-composition can be identified by the number and found in Supplementary Table S4

Unsupervised PCA shows some degree of clustering based on histology type, NCE, cancer, or stroma (Fig. 1). According to both PCA models (negative and positive ion mode), NCE and cancer cluster closer together, while stroma is a slightly more separate cluster. Some clustering was additionally observed for patient origin as shown in Supplementary Figure S1, which also show the PCA loading plots. The six different supervised OPLS-DA models, pairwise comparing the three different tissue types in both ion mode data sets, all showed significant differentiation after permutation testing (*p* < 0.001) with prediction accuracies ranging from 71.9 to 87.1% (Supplementary Table S3). The first latent variable in each OPLS-DA model represents the variance showing the best separation between the tissue types, and the VIP-score was calculated to determine discriminatory importance of each variable, both of which are plotted for each model in Fig. 2. Variables with VIP score > 1 were considered important for separation. Scores plots are presented in Supplementary Figure S2, and VIP scores for all masses are reported in Supplementary Table S5 and S6. These OPLS-DA models reveal several metabolic alterations between the different tissue types in our prostate tissue samples. Additionally, univariate LMM supported the finding of several masses being significantly different between the tissue types (Supplementary Table S5 and S6). Identified metabolites and lipids with significantly different levels between NCE and cancer are shown in Table 1.

**Table 1:** Identified and significantly different masses between cancer and non-cancer epithelium after univariate linear mixed models testing. Log_2_ fold change (Log_2_FC) is reported comparing cancer to non-cancer epithelium. ‘ID in OPLS-DA’ can be used to find the analyte on the loadings plot in Fig. 2. The reported *m/z* values are from MALDI-TOF measurements. LPC = lysophospatidylcholine, PE = phosphatidylethanolamine, and PS = phosphatidylserine

*m/z* MALDI-TOF	ID	ID in OPLS-DA	Log_2_FC	*p* value
104.17	Choline	Choline	0.785	9.6 × 10^−4^
132. 03	Aspartate	Aspartate	− 0.579	2.5 × 10^−6^
146.17	Acetylcholine	ACho	0.537	5.1 × 10^−4^
162.16	Carnitine	Carnitine	0.747	2.3 × 10^−9^
167.03	Urate	Urate	0.290	2.2 × 10^−3^
174.83*	Zinc (ZnCl_3_^−^)	Zinc	− 1.054	1.9 × 10^−4^
175.02**	Ascorbate/Glucurone	Ascorbate	− 0.281	0.010
191.02	Citrate	Citrate	− 0.892	2.3 × 10^−7^
204.17	Acetylcarnitine	Acetylcarnitine	0.789	1.7 × 10^−4^
248.19	Hydroxybutyrylcarnitine	HBCt	0.888	1.0 × 10^−3^
426.01	Adenosine diphosphate	ADP	0.387	0.025
518.37	LPC (16:0)	LPC 82	− 1.384	7.8 × 10^−6^
534.34	LPC (16:0)	LPC 86	− 1.168	1.6 × 10^−5^
544.39	LPC (18:1)	LPC 90	− 0.744	1.0 × 10^−4^
792.53	PE (20:1_20:4)	PE 112	0.590	0.041
838.51	PS (40:4)	PS 124	0.326	8.8 × 10^−3^

*For ZnCl_3_^-^, the isotopes with *m/z* 174.83 were used to represent zinc due to an overlapping contaminant [17]

**Ascorbate and glucurone have identical masses and MS/MS suggests a mix of the two metabolites, which both belongs to the same Ascorbate and aldarate metabolic pathway

### Metabolites associated with healthy prostate function have reduced levels in cancer tissue

**Fig. 3:**
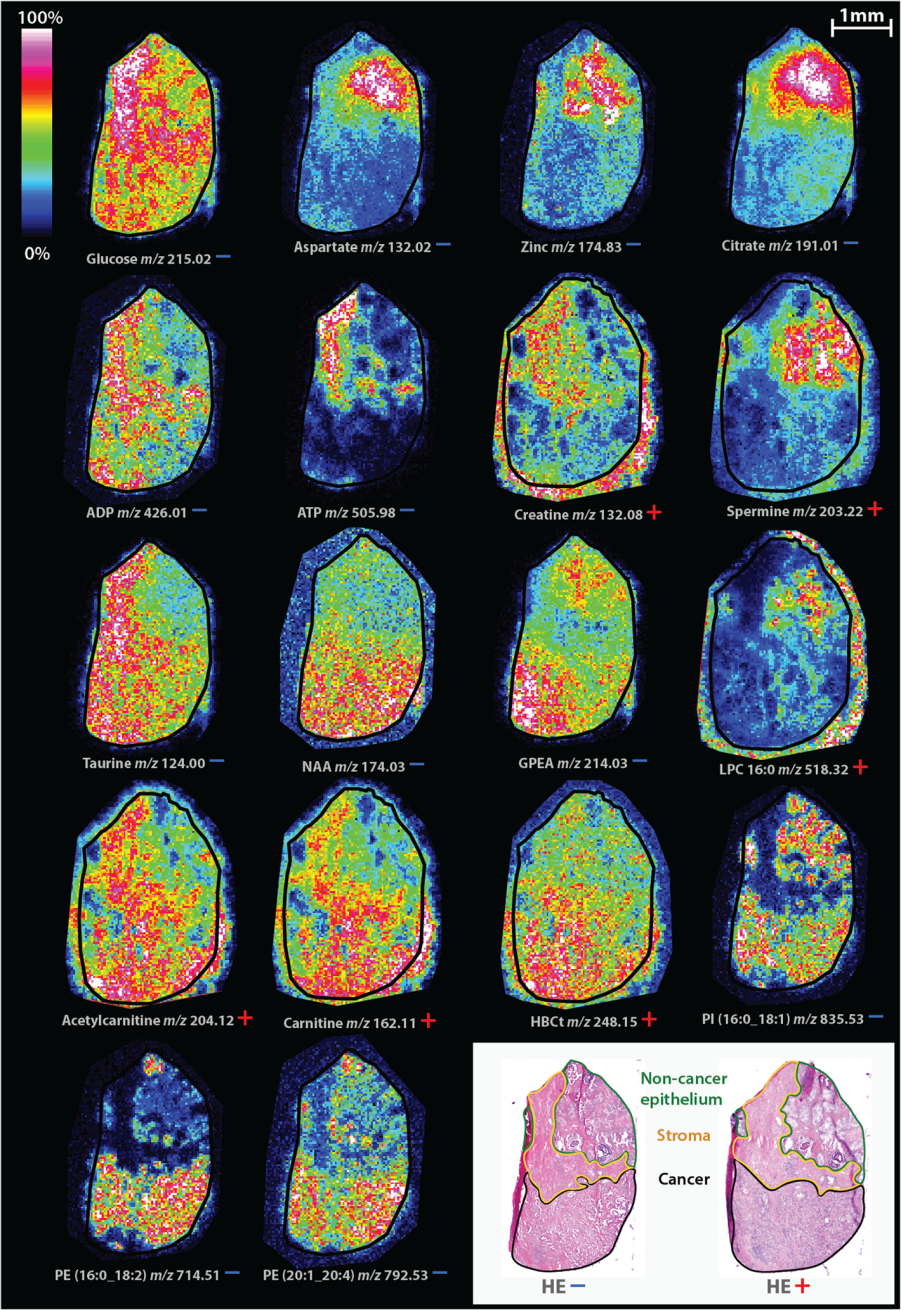
Spatial distribution of identified masses in both ion modes on consecutive tissue sections. Masses detected in positive and negative ion mode are indicated with a red plus and blue minus sign, respectively. These sections derive from patient nr. 11 (Supplementary Table S1). For clarity, tissue edges are outlined with a black border. Note that the cancer region also contains some stroma finely mixed with cancer cells that could not be annotated separately. ADP = adenosine diphosphate, ATP = adenosine triphosphate, GPEA = glycerylphosphorylethanolamine, HBCt = hydroxybutyrylcarnitine, HE = hematoxylin and eosin, LPC = lysophosphatidylcholine, NAA = *N*-acetylaspartate, PE = phosphatidylethanolamine, PI = phosphatidylinositol

We found citrate, aspartate, and zinc, which are mechanistically closely related to have lower levels in cancer tissue compared to NCE through both OPLS-DA and LMM modeling (Fig. 2c and Fig. 3, Table 1), similar to our recent publication [17]. Citrate, aspartate, zinc, and the polyamine spermine are typical metabolic actors with particular high levels in the healthy prostate epithelium [11, 33]. Reduced levels of these metabolites are repeatedly reported in prostate cancer tissue [12, 14, 34]. The polyamine spermine had reduced levels in cancer compared to NCE in the OPLS-DA model (Fig. 2d and Fig. 3, log_2_FC = −0.657, VIP = 1.20) and was close to significant in LMM (*p* = 0.061). In contrast, the other polyamine identified, spermidine, had a slightly, but not significant, higher level in cancer (log_2_FC = 0.259, VIP = 0.42, *p* = 0.45).

### Metabolic profiling of prostate stroma shows higher levels of taurine, glucose, creatine, AMP, ADP, and ATP

As expected, metabolites associated with healthy prostatic epithelial function (citrate, aspartate, zinc and spermine) had lower levels in stroma compared to NCE (Fig. 2a, b, Supplementary Table S5 and S6). Stroma also tended to have lower levels of phospholipids compared to both cancer and NCE (Fig. 2a, b, e, f). Taurine, which among other roles functions as an antioxidant, had a significantly higher level in stroma compared to NCE (log_2_FC = 0.516, *p* = 1.6 × 10^−9^, VIP = 1.11, Fig. 2a) and also showed close to significantly higher levels in stroma compared to cancer (log_2_FC = 0.514, *p* = 0.078, VIP = 1.09, Fig. 2e). Glucose also had significantly higher levels in stroma compared to both NCE (log_2_FC = 0.306, *p* = 1.6 × 10^−3^, VIP = 0.76) and cancer (log_2_FC = 0.533, *p* = 0.024, VIP = 1.10). It should be noted that it is not possible to differentiate glucose from other sugars of similar mass using MS/MS, but based on a previous publication, most of the *m/z* 215.03 sugars on tissue is glucose [2]. The LMM tests identified significantly higher levels of the nucleotides, AMP, ADP, and ATP, in stroma. With the exception of AMP between stroma and cancer (*p* = 0.065), this was the case for all adenosine phosphates when comparing stroma to both NCE and cancer (*p* < 0.05). Lastly, there were higher levels of creatine in stroma compared to NCE (log_2_FC = 0.431, *p* = 1.4 × 10^−4^, VIP = 0.90) and cancer (log_2_FC = 0.583, *p* = 0.15, VIP = 1.15).

### Higher levels of carnitine shuttle metabolites in cancer tissue

**Fig. 4:**
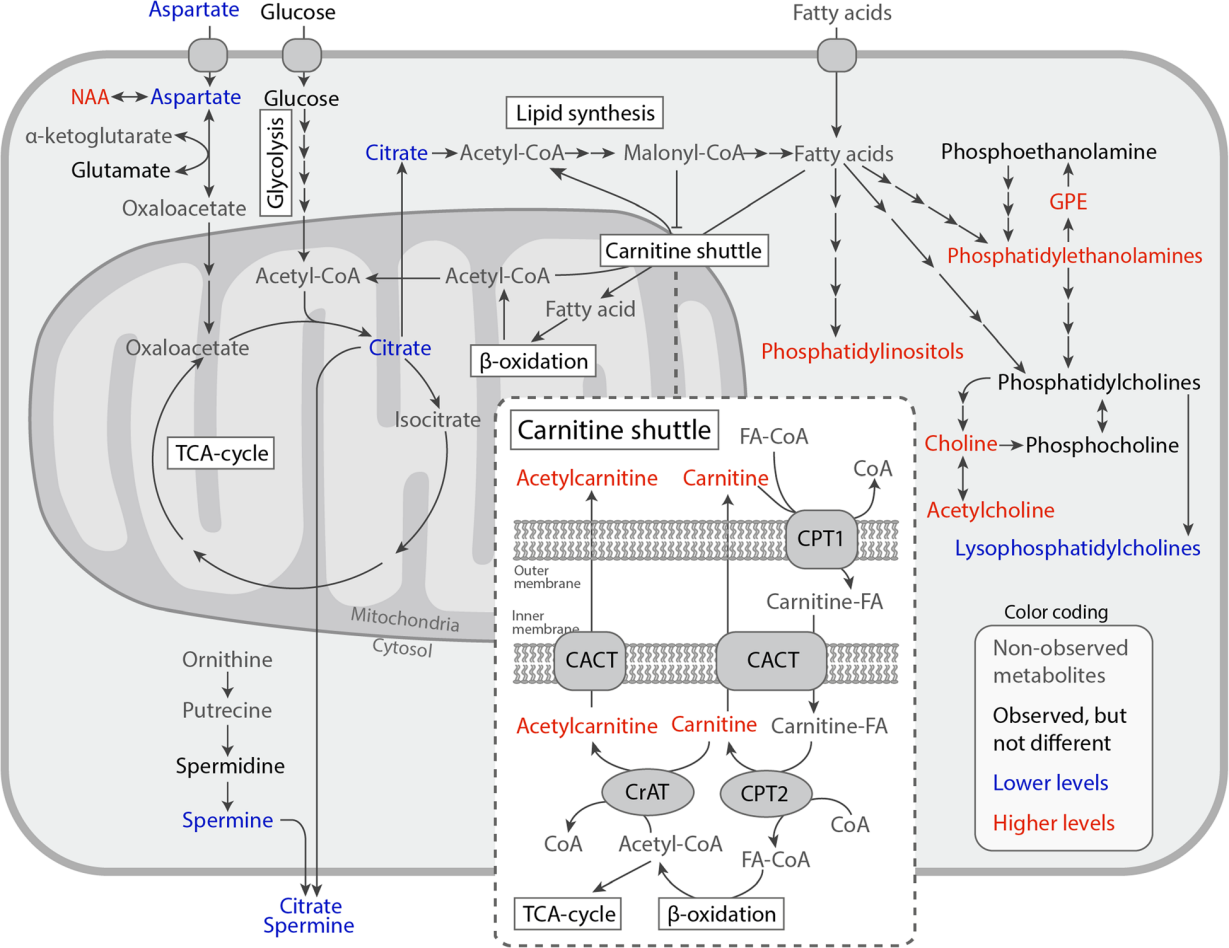
Altered metabolic pathways in cancer compared to non-cancer epithelium (NCE). Metabolites and lipids with variable importance on the projection (VIP) score > 1 in the orthogonalized partial least squares discrimination analysis (OPLS-DA) models comparing NCE to cancer, are colored as red or blue for higher or lower levels in cancer, respectively. CoA = coenzyme A, CPT1 = carnitine palmitoyltransferase 1, CACT = carnitine-acetylcarnitine transferase, CrAT = carnitine O-acetyl-transferase, FA = fatty acid, GPE = glycerophosphoethanolamine, and NAA = *N*-acetylaspartate

Carnitine and acetylcarnitine were significantly upregulated in cancer compared to NCE (VIP ≥ 1.93, *p* < 0.001, Fig. 2d, Fig. 3, and Table 1), in addition to borderline significant higher levels compared to stroma (VIP = 1.26, *p* ≤ 0.076, Fig. 2f). Carnitine and acetylcarnitine are two crucial metabolites functioning together in the carnitine shuttle, which transports fatty acid groups across the mitochondrial membrane for energy production through β-oxidation (Fig. 4) [35]. Two other carnitine species were also identified in this dataset, hydroxybutyrylcarnitine (HBCt) and butyryl-L-carnitine, with HBCt having significant elevation in cancer compared to NCE and stroma (Fig. 2d, f, log_2_FC > 0.823, *p* ≤ 0.001, VIP > 1.18).

### Altered lipid metabolism in cancer tissue

Several metabolites related to lipid synthesis and lipid rearrangement had higher levels in cancer compared NCE, including choline (log_2_FC = 0.785, *p* = 9.6 × 10^−4^, VIP = 2.04), acetylcholine (log_2_FC = 0.537, *p* = 5.1 × 10^−4^, VIP = 1.48), *N*-acetylaspartate (NAA, log_2_FC = 0.734, *p* = 0.22, VIP = 1.59), and glycerophoshoethanolamine (GPE, log_2_FC = 0.818, *p* = 0.062, VIP = 1.2). A few other compounds involved in lipid synthesis, phosphocholine, glycerophosphocholine, and phosphoethanolamine, did not have significantly higher levels in cancer tissue (Fig. 2c, d, Supplementary Table S5 and S6).

Increased lipid synthesis is also showcased by higher levels of many of its end-products, the phospholipids. Particularly phosphatidylethanolamines (PE) and phosphatidylinositol (PI) measured in negative ion mode had higher levels in cancer tissue compared to NCE in the OPLS-DA models (VIP > 1, Fig. 2c). In contrast, all identified lysophospatidylcholines (LPC) had significantly reduced levels in cancer compared to NCE (log_2_FC ≤ − 0.744, *p* < 0.001, VIP ≥ 1.91, Fig. 2d, Table 1).

## Discussion

This study demonstrates clear differences in metabolism associated with the tissue types, NCE, stroma, and cancer in prostate tissue samples. Performing MSI in both positive and negative ion mode on adjacent sections, allowed us to detect and identify a wider range of chemically different metabolites and lipids than previous studies using MSI to investigate prostate tissue [4, 15, 16, 23]. Altered metabolic pathways found between cancer and NCE are summarized in Fig. 4.

Stroma tissue mainly consists of smooth muscle cells in addition to a smaller number of fibroblasts and immune cells and has different functions and metabolic profiles than epithelial cells. During prostate cancer progression, the volume of stromal components decreases as cancer cells invade more space in the tissue. Hence, the percentage of stroma content is not balanced between normal and cancer samples, making stroma a confounding factor for prostate tissue bulk analysis [1]. Previous MSI analysis of prostate tissue have either excluded stroma tissue from data analysis [4, 15, 23] or combined stroma tissue together with normal epithelial tissue [3]. In the latter case, observed metabolic differences between cancer and normal tissue can be a result of reduced presence of stroma in cancer tissue rather than a difference between cancer and healthy glands. We therefore found it important to investigate the metabolic profile of the often over-looked stroma tissue. Our unsupervised PCA analysis shows that metabolic measurements from stroma cluster more separately compared to both NCE and cancer measurements (Fig. 1), demonstrating that stroma is a tissue type with a distinct molecular profile. Noteworthy, the antioxidant taurine, reported to have elevated levels at inflammatory sites [36], was increased in stroma according to our supervised analysis (Fig. 2a, c, e). Cell studies also suggest that taurine may have pro-apoptotic and anti-tumor effects [37]. Alterations in taurine levels are not widely reported for prostate cancer tissue samples and a study previously published by our group did not identify any change in taurine levels when comparing prostate cancer to benign tissue samples using whole-sample nuclear magnetic resonance (NMR) measurements [12]. We did, however, identify higher levels of taurine in the same data set when investigating reactive stroma, along with considerable upregulation of immune related genes [38]. Reactive stroma is generally characterized by inflammation and extracellular matrix remodeling [39, 40]. Collectively, our findings suggest that taurine is primarily present in the stroma, potentially as a protective reaction to a more stressful environment caused by inflammation and oxidative stress [41]. Future investigation of reactive prostate stroma using MSI may give more information on taurine’s role in stroma.

A key characteristic of the prostate stroma is the high content of smooth muscle cells. The observation that stroma had the highest levels of the nucleotides, AMP, ADP, and ATP, may be explained by the presence of muscle cells. ATP is the main energy currency for all cells, particularly in muscle cells where hydrolysis of phosphate groups with the end products ADP and AMP, is important for muscle contraction [42]. Hence, the muscle cells of the prostate stroma may contain a larger reservoir of these adenosine phosphates to facilitate fast contraction of the prostate when needed, e.g., during ejaculation. Further, glucose, an important source for ATP production, and creatine, a common storage molecule for phosphate groups in muscle cells [42], had higher levels in stroma compared to both NCE and cancer. Our metabolic profiling of prostate stroma clearly reveals pathways related to its muscle contraction functionality.

Citrate and zinc were among the most important variables to differentiate NCE and cancer, which had decreased levels in cancer. Reduced levels of citrate in prostate cancer tissue is a widely reported metabolic alteration [12, 13] and have also been shown through MSI experiments using DESI [15, 16]. The citrate level is closely associated with zinc, which is suggested to cause accumulation of citrate by inhibiting utilization in the TCA cycle [11], as well as aspartate, which functions as a carbon source for citrate synthesis (Fig. 4) [43]. Recently, we were for the first time able to simultaneously detect the metal zinc together with metabolites using MALDI MSI, further demonstrating the close metabolic association and reduced levels in cancer [17].

The polyamine spermine, which similar to citrate, is a metabolite produced and secreted by the healthy prostate, had lower levels in cancer compared to NCE (Fig. 2d and Fig. 3). To our knowledge, this is the first time spermine is detected with MSI on human tissue. Although many have shown reduced levels of spermine in prostate cancer [12, 13, 44], less is known about the metabolic mechanism behind this change. There is reported increased expression of spermine oxidase (enzyme that converts spermine to spermidine) in prostate cancer [45], which could explain the slight elevation of spermidine shown in this study and by others [34].

Increased de novo lipid synthesis for membrane production is a key feature of many cancer types, including prostate cancer [10]. In this study, small metabolites involved in phospholipid synthesis and rearrangement, such as choline and GPE, had higher levels in cancer compared to NCE. Choline is a crucial building block for PC synthesis. Elevated GPE in cancer tissue also gives evidence towards membrane breakdown and rebuilding. GPE is a breakdown product of PEs [46] and can be further broken down to phosphoethanolamine that can be used for new phospholipid synthesis of PE and sphingolipids (Fig. 4). Higher levels of GPE in prostate cancer compared to benign hyperplasia have been reported [22]. We also detected elevated levels of phospholipids in cancer compared to NCE, particularly several PE and PI in negative ion mode. In contrast, there were no remarkable differences of either the intermediates phosphocholine, glycerophosphocholine, ethanolamine, or PCs between cancer and NCE. This is an unexpected observation, as these metabolites and lipids usually have higher levels in prostate cancer [22]. Further investigation of ethanolamine and choline containing compounds detection with MALDI is required to solve this discrepancy. LPCs had lower levels in cancer compared to NCE. In particular, LPC(16:0) had the highest VIP score (2.26) in the OPLS-DA model comparing cancer to NCE and were highly significant (*p* = 7.8 × 10^−6^). This is in accordance with Goto et al. which also identified LPC(16:0) as reduced in prostate cancer using MALDI imaging, and further found that it was significantly associated with biochemical recurrence after radical prostatectomy [4]. LPCs may therefore be useful clinical biomarkers but needs verification in a larger patient cohort.

Increased β-oxidation, the process in which fatty acids are broken down for energy production, is another metabolic alteration commonly detected in cancer [35]. The rate limiting step of β-oxidation is the carnitine shuttle, which transports fatty acids across the mitochondrial membranes by attaching them to the metabolite carnitine and creating carnitine fatty acid (carnitine-FA) [35]. Excess acetyl groups inside the mitochondria can also be transported out by attaching them to carnitine, creating acetylcarnitine. The elevated levels in prostate cancer of carnitine and acetylcarnitine, as well as hydroxybutyrylcarnitine, provide evidence for elevated β-oxidation. Ren et al. showed elevated levels of both carnitine and several carnitine-FA in prostate cancer compared to normal tissue samples [47], and Randall et al. detected a higher intensity of palmitoylcarnitine in prostate cancer areas using MALDI MSI [19]. Recently, higher levels of carnitines were detected in prostate cancer tissue with presence of the gene fusion *TMPRSS2-ERG* (transmembrane protease, serine 2—ETS-related gene), a marker associated with shorter recurrence-free survival and cancer specific death [48]. Increased levels of carnitines can also be non-invasively detected in serum [49, 50] and extracellular vesicles [51] of patients with aggressive prostate cancer. Further, the enzymes that transport fatty acids through the carnitine shuttle (Fig. 4) are upregulated in prostate cancer tissue [18, 47, 52]. Collectively, the carnitine shuttle system has an interesting potential to be investigated further for diagnostics.

There is a limitation in this study which should be addressed. Delocalization of metabolites to the outside of the tissue’s boundary was observed for the sections measured in positive ion mode (Fig. 3), which is an indication of delocalization also within the tissue. This is likely an effect of insufficient vacuum drying and/or a wet matrix application. Due to the nature of diffusion, with molecules moving from high to low concentrations, delocalization could cause false negatives by decreasing the signal-differences between tissue types but is less likely to cause false positives.

MSI methods show potential for diagnostic applications and are getting increasingly intertwined within clinics [53]. Several of the differential analytes identified in this study may be good candidates for clinical biomarkers, as they have also been reported differential in other publications using MSI, including citrate [15, 16], and LPC (16:0) [4]. Importantly, future studies should aim to link MALDI MSI measurements to clinical follow-up data such as recurrence, metastasis, and overall survival. However, before MSI can be routinely implemented in the clinic, there is a need to further understand tissue heterogeneity and to improve multicenter reproducibility and validate biomarker accuracy [53].

## Conclusion

This study shows that different tissue entities within prostate cancer tissue samples have distinct metabolic profiles. We identified significant metabolic alterations in key molecular processes, such as lipid metabolism and prostatic secretory function between the tissue types NCE, stroma, and cancer, using MALDI-TOF MSI. Profiling of stroma revealed higher levels of energy transfer metabolites and the antioxidant taurine compared to cancer and NCE. An interesting finding was elevated levels of key carnitine shuttle metabolites in prostate cancer tissue compared to both NCE and stroma, providing evidence for elevated lipid β-oxidation. The observed differences in metabolite levels between the defined tissue structures pinpoint the importance of methodology providing spatial information. In a clinical setting, capturing this spatial information in heterogeneous cancer samples provides a potentially vital advantage over bulk analysis, where important differential biomarker levels may be hidden.

## Supplementary Information

**Additional file 1: Table S1.** Clinical data of prostate cancer patient donors at the time of surgery and tissue collection. **Table S2.** Parameters used for spraying matrices 2,5-dihydroxybenzoic acid (DHB) and N-(1-naphthyl) ethylenediamine dihydrochloride (NEDC) with HTX TM-SprayerTM. **Table S3.** Overview of number of spectra and multivariate orthogonal partial least squares discriminant analysis (OPLS-DA) models. **Table S4.** Mass identification through MS/MS and accurate mass of parent ion. PE = phosphoethanolamines, PC = phosphatidylcholines, PG = phosphatidylglycerols, PI = phosphatidylinositols, PS = phosphatidylserine and SM = sphingomyelin. **Table S5.** Full peak list for masses in negative ion mode with NEDC matrix. Table includes mean peak height and standard deviation (SD) across different tissue types. Non-cancer epithelium (NCE), stroma and cancer were pairwise compared and here we present log^2^ fold change (Log_2_FC), adjusted *p*-values from linear mixed models and variable importance on the projection (VIP) scores from orthogonal projections to latent structures discriminant Analysis (OPLS-DA) models. **Table S6.** Full peak list for masses in positive ion mode with DHB matrix. Table includes mean peak height and standard deviation (SD) across different tissue types. Non-cancer epithelium (NCE), stroma and cancer were pairwise compared and here we present log_2_ fold change (Log_2_FC), adjusted p-values from linear mixed models and variable importance on the projection (VIP) scores from orthogonal projections to latent structures discriminant Analysis (OPLS-DA) models. **Figure S1.** Principal component analysis (PCA) of metabolites and lipids detected with MALDI MSI. **Figure S2**. Score plots for orthogonalized partial least squares discrimination analysis (OPLS-DA) models. 

## Data Availability

Processed data used for all statistical methods in this paper can be accessed from Figshare (10.6084/m9.figshare.c.5140679.v1). Due to data size and privacy regulations, the raw data that support the findings of this study are available from the corresponding authors upon request.

## Abbreviations

ACho: Acetylcholine
ADP: Adenosine diphosphate
AMP: Adenosine monophosphate
ATP: Adenosine triphosphate
DHB: 2,5-dihydroxybenzoic acid
FA: Fatty acid
FAS: Fatty acid synthesis
GPE: Glycerophoshoethanolamine
H&E: Hematoxylin and eosin
HBCt: Hydroxybutyrylcarnitine
ITO: Indium tin oxide
LA-ICP: Laser ablation inductively coupled plasma
LMM: Linear mixed models
LPC: Lysophosphocholine
MALDI: Matrix assisted laser desorption/ionization
MS/MS: Tandem mass spectrometry
MSI: Mass spectrometry imaging
NAA: *N*-acetylaspartate
NCE: Non-cancer epithelium
NEDC: *N*-(1-naphthyl) ethylenediamine dihydrochloride
NMR: Nuclear magnetic resonance
OPLS-DA: Orthogonal partial least squares discriminant analysis
PC: Phosphatidylcholines
PCA: Principal component analysis
PE: Phosphatidylethanolamines
PEA: Phosphoethanolamine
PG: Phosphatidylglycerols
PI: Phosphatidylinositols
PS: Phosphatidylserine
SM: Sphingomyelin
TOF: Time-of-flight
VIP: Variable importance on the projection
