# Supplementary material for: Spatial differentiation of metabolism in prostate cancer tissue by MALDI-TOF MSI
Source: Cancer Metab. 2021 Jan 29;9:9. doi: 10.1186/s40170-021-00242-z (PMC7847144; doi:10.1186/s40170-021-00242-z)
Supplement: Supplementary file 1 — Additional file 1: Table S1. Clinical data of prostate cancer patient donors at the time of surgery and tissue collection. Table S2. Parameters used for spraying matrices 2,5-dihydroxybenzoic acid (DHB) and N-(1-naphthyl) ethylenediamine dihydrochloride (NEDC) with HTX TM-SprayerTM. Table S3. Overview of number of spectra and multivariate orthogonal partial least squares discriminant analysis (OPLS-DA) models. Table S4. Mass identification through MS/MS and accurate mass of parent ion. PE = phosphoethanolamines, PC = phosphatidylcholines, PG = phosphatidylglycerols, PI = phosphatidylinositols, PS = phosphatidylserine and SM = sphingomyelin. Table S5. Full peak list for masses in negative ion mode with NEDC matrix. Table includes mean peak height and standard deviation (SD) across different tissue types. Non-cancer epithelium (NCE), stroma and cancer were pairwise compared and here we present log2 fold change (Log2FC), adjusted p-values from linear mixed models and variable importance on the projection (VIP) scores from orthogonal projections to latent structures discriminant Analysis (OPLS-DA) models. Table S6. Full peak list for masses in positive ion mode with DHB matrix. Table includes mean peak height and standard deviation (SD) across different tissue types. Non-cancer epithelium (NCE), stroma and cancer were pairwise compared and here we present log2 fold change (Log2FC), adjusted p-values from linear mixed models and variable importance on the projection (VIP) scores from orthogonal projections to latent structures discriminant Analysis (OPLS-DA) models. Figure S1. Principal component analysis (PCA) of metabolites and lipids detected with MALDI MSI. Figure S2. Score plots for orthogonalized partial least squares discrimination analysis (OPLS-DA) models. [file 40170_2021_242_MOESM1_ESM.pdf]

Supplementary Information to:

Spatial differentiation of metabolism in prostate cancer tissue by MALDI-TOF MSI

Maria K. Andersen[1]*, Therese S. Høiem[1], Britt S.R. Claes[2], Benjamin Balluff[2], Marta Martin-Lorenzo[2], Elin Richardsen[3,4], Sebastian Krossa[1], Helena Bertilsson[5,6], Ron M.A. Heeren[2], Morten B. Rye[5,7-9], Guro F. Giskeødegård[1], Tone F. Bathen[1], May-Britt Tessem[1,7]*

[1] *Department of Circulation and Medical Imaging, NTNU - Norwegian University of Science and Technology, Trondheim, Norway.*
[2] *Maastricht MultiModal Molecular Imaging institute (M4I), Maastricht University, Maastricht, The Netherlands.*
[3] *Department of Medical Biology, UiT The Artic University of Norway, Tromsø, Norway.*
[4] *Department of Clinical Pathology, University Hospital of North Norway, UNN Tromsø, Norway.*
[5] *Department of Clinical and Molecular Medicine, NTNU - Norwegian University of Science and Technology, Trondheim, Norway*
[6] *Department of Urology, St. Olavs Hospital, Trondheim University Hospital, Trondheim, Norway.*
[7] *Clinic of Surgery, St. Olavs Hospital, Trondheim University Hospital, Trondheim, Norway.*
[8] *Clinic of Laboratory Medicine, St.Olavs Hospital, Trondheim University Hospital, Trondheim, Norway*
[9] *BioCore - Bioinformatics Core Facility, NTNU – Norwegian University of Science and Technology, Trondheim, Norway*

*Corresponding authors
Maria K. Andersen: maria.k.andersen@ntnu.no
May-Britt Tessem: may-britt.tessem@ntnu.no

Supplementary Table S1: Clinical data of prostate cancer patient donors at the time of surgery and tissue collection.

Patient nr.	Age	T-stage	Grade Group	Pre-operative PSA
1	62	T2*	4	8.0
2	61	T3a	5	21.4
3	63	T3b	5	6.4
4	48	T2c	3	10.4
5	56	T2c	2	10.7
6	61	T2c	2	14.5
7	69	T3a	3	9.0
8	68	T3b	5	14.6
9	67	T3b	5	13.2
10	61	T2c	2	5.2
11	69	T3a	5	5.6
12	66	T2c	3	15.3
13	66	T2c	2	6.9
14	69	T2c	3	12.0
15	69	T3b	5	13.1

*Extent of tumor within the prostate capsule is unknown

Supplementary Table S2: Parameters used for spraying matrices 2,5-dihydroxybenzoic acid (DHB) and N-(1-naphthyl) ethylenediamine dihydrochloride (NEDC) with HTX TM-Sprayer^{TM}

Parameters / Matrix	DHB	NEDC
Temperature of spray	85 °C	30 °C
Passes	10	14
Flow rate	0.11 ml/min	0.06 ml/min
Velocity	1200 mm/min	1200 mm/min
Track spacing	3 mm	3 mm
Spray movement pattern	CC	CC
Pressure	10 psi	10 psi
Gas flow rate	3 l/min	2 l/min
Drying time	0 min	0 min
Nozzle Ht	40 mm	40 mm

Supplementary Table S3: Overview of number of spectra and multivariate orthogonal partial least squares discriminant analysis (OPLS-DA) models.

	Negative ion mode	Positive ion mode
**Number of spectra:**		
Total	188 123	188 440
Non-cancer epithelium	37 662	41 685
Cancer	64 263	65 997
Stroma	86 198	80 758
**Variables/Features**	136	167
Multivariate OPLS-DA models		
**Non-cancer epithelium vs. stroma:**		
Prediction accuracy	79.9 %	81.1 %
Sensitivity	72.7 %	72.6 %
Specificity	87.0 %	89.5 %
**Non-cancer epithelium vs. cancer:**		
Prediction accuracy	71.9 %	78.4 %
Sensitivity	81.2 %	83.9 %
Specificity	62.7 %	72.9 %
**Cancer vs. stroma:**		
Prediction accuracy	83.8 %	87.1 %
Sensitivity	90.2 %	83.9 %
Specificity	77.5 %	83.3 %

Supplementary Table S4: Mass identification through MS/MS and accurate mass of parent ion. PE = phosphoethanolamines, PC = phosphatidylcholines, PG = phosphatidylglycerols, PI = phosphatidylinositols, PS = phosphatidylserine and SM = sphingomyelin.

m/z value	Assignment	ID in OPLS-DA loadings plot	Adduct	ppm error	NCE HCD
104.107	Choline	Choline	[M]+	-0.294	80
124.00737	Taurine	Taurine	[M-H]-	-0.138	60
132.0303	Aspartate	Aspartate	[M-H]-	0.523	10
132.07679	Creatine	Creatine	[M+H]+	0.507	30
140.01186	Phosphoethanolamine	Phosphoethanolamine	[M-H]-	0.303	30
145.0619	Glutamine	Glutamine	[M-H]-	0.238	40
146.04592	Glutamate	Glutamate	[M-H]-	0.267	40
146.11752	Acetylcholine	ACho	[M+H]+	-0.105	55
146.16517	Spermidine	Spermidine	[M+H]+	-0.029	55
162.11236	Carnitine	Carnitine	[M+H]+	0.124	65
167.021	Urate	Urate	[M-H]-	-0.618	40
174.04092	*N*-Acetylaspartate	NAA	[M-H]-	0.715	20
174.8292*	Zinc ($ZnCl_3^-$)	Zinc	[M+3Cl]-	1.144	20
175.0247**	Ascorbate/Glucuronolactone	Ascorbate	[M-H]-	-0.578	35
184.0734	Phosphocholine	Phosphocholine	[M+H]+	0.158	55
191.01996	Citrate	Citrate	[M-H]-	1.226	35
203.22316	Spermine	Spermine	[M+H]+	0.426	30
204.12327	Acetylcarnitine	Acetylcarnitine	[M+H]+	-0.071	35
214.04895	Glycerophosphorylethanolamine	GPE	[M-H]-	1.650	45
215.03299	Glucose	Glucose	[M+Cl]-	0.934	40
232.15421	Butyryl-L-carnitine	BCt	[M+H]+	0.368	40
248.14909	Hydroxybutyrylcarnitine	HBCt	[M+H]+	0.164	30
280.09182	Glycerophosphocholine	GPC	[M+Na]+	0.054	35
306.07678	Glutathione	GSH	[M-H]-	0.819	28
346.05596	Adenosine monophosphate	AMP	[M-H]-	0.441	–
426.02268	Adenosine diphosphate	ADP	[M-H]-	1.273	–
505.98866	Adenosine triphosphate	ATP	[M-H]-	0.379	–
518.32192	LPC (16:0)	LPC 82	[M+Na]+	-0.541	25
534.29597	LPC (16:0)	LPC 86	[M+K]+	-0.557	25
544.33829	LPC (18:1)	LPC 90	[M+Na]+	0.807	25
714.50877	PE (16:0_18:2)	PE 91	[M-H]-	1.179	25
716.52449	PE 16:0_18:1)	PE 92	[M-H]-	1.273	25
725.55668	SM (34:1;2)	SM 125	[M+Na]+	-0.160	25
738.50883	PE (16:0_20:4)	PE 94	[M-H]-	1.222	25
740.52458	PE (36:3)	PE 95	[M-H]-	1.353	25
742.5402	PE (18:1_18:1)	PE 96	[M-H]-	1.309	25
744.55594	PE (18:0_18:1)	PE 97	[M-H]-	1.426	25
747.5191	PG (16:1_18:0)	PG 98	[M-H]-	1.26	25
756.55124	PC (16:0_16:0)	PC 135	[M+Na]+	-0.180	25
760.58464	PC (16:0_18:1)	PC 137	[M+H]+	-0.633	25
762.50981	PE (16:0_22:6)	PE 103	[M-H]-	2.468	25
764.52525	PE (18:1_20:4)	PE 104	[M-H]-	2.187	25
766.54013	PE (18:0_20:4)	PE 105	[M-H]-	1.177	25
770.57241	PE (18:1_20:1)	PE 106	[M-H]-	2.442	25
772.52517	PC (32:0)	PC 140	[M+K]+	-0.185	25
780.55117	PC (16:0_18:2)	PC 142	[M+H]+	-0.264	25
782.56709	PC (16:0_18:1)	PC 143	[M+Na]+	0.082	25
786.53096	PS (36:2)	PS 109	[M-H]-	2.419	25
788.54575	PS (18:0_18:1)	PS 110	[M-H]-	1.322	23
792.55683	PE (20:1_20:4)	PE 112	[M-H]-	2.463	25
798.54063	PC (34:1)	PC 146	[M+K]+	-0.780	25

804.55131	PC (36:4)	PC 147	[M+Na]+	-0.082	25
808.58272	PC (36:2)	PC 149	[M+Na]+	0.054	25
810.59933	PC (36:1)	PC 150	[M+Na]+	1.238	25
812.54675	PS (18:0_20:3)	PS 118	[M-H]-	2.514	23
816.57836	PS (38:1)	PS 120	[M-H]-	2.881	20
820.52523	PC (36:4)	PC 153	[M+K]+	-0.101	25
822.54091	PC (36:3)	PC 154	[M+K]+	-0.065	25
824.55656	PC (36:2)	PC 155	[M+K]+	-0.065	25
826.57235	PC (36:1)	PC 156	[M+K]+	0.105	25
832.58254	PC (38:4)	PC 158	[M+Na]+	-0.163	25
833.52092	PI (16:0_18:2)	PI 121	[M-H]-	2.841	32
834.53142	PS (18:0_22:6)	PS 122	[M-H]-	2.831	25
835.53596	PI (16:0_18:1)	PI 123	[M-H]-	2.104	32
838.56245	PS (40:4)	PS 124	[M-H]-	2.496	20
844.6091	PS (18:1_22:0)	PS 125	[M-H]-	2.276	22
848.55647	PC (38:4)	PC 163	[M+K]+	-0.169	25
850.57256	PC (38:3)	PC 164	[M+K]+	0.349	25
857.52048	PI (16:0_20:4)	PI 126	[M-H]-	2.248	30
861.55173	PI (18:1_18:1)	PI 128	[M-H]-	2.18	33
863.56757	PI (18:0_18:1)	PI 129	[M-H]-	2.394	32

*For $ZnCl_3^-$, the isotopes with *m/z* 174.83 were used to represent zinc due to an overlapping contaminant.
** Ascorbate and glucurone have identical masses, and MS/MS suggests a mix of the two metabolites, which both belongs to the same Ascorbate and aldarate metabolic pathway.

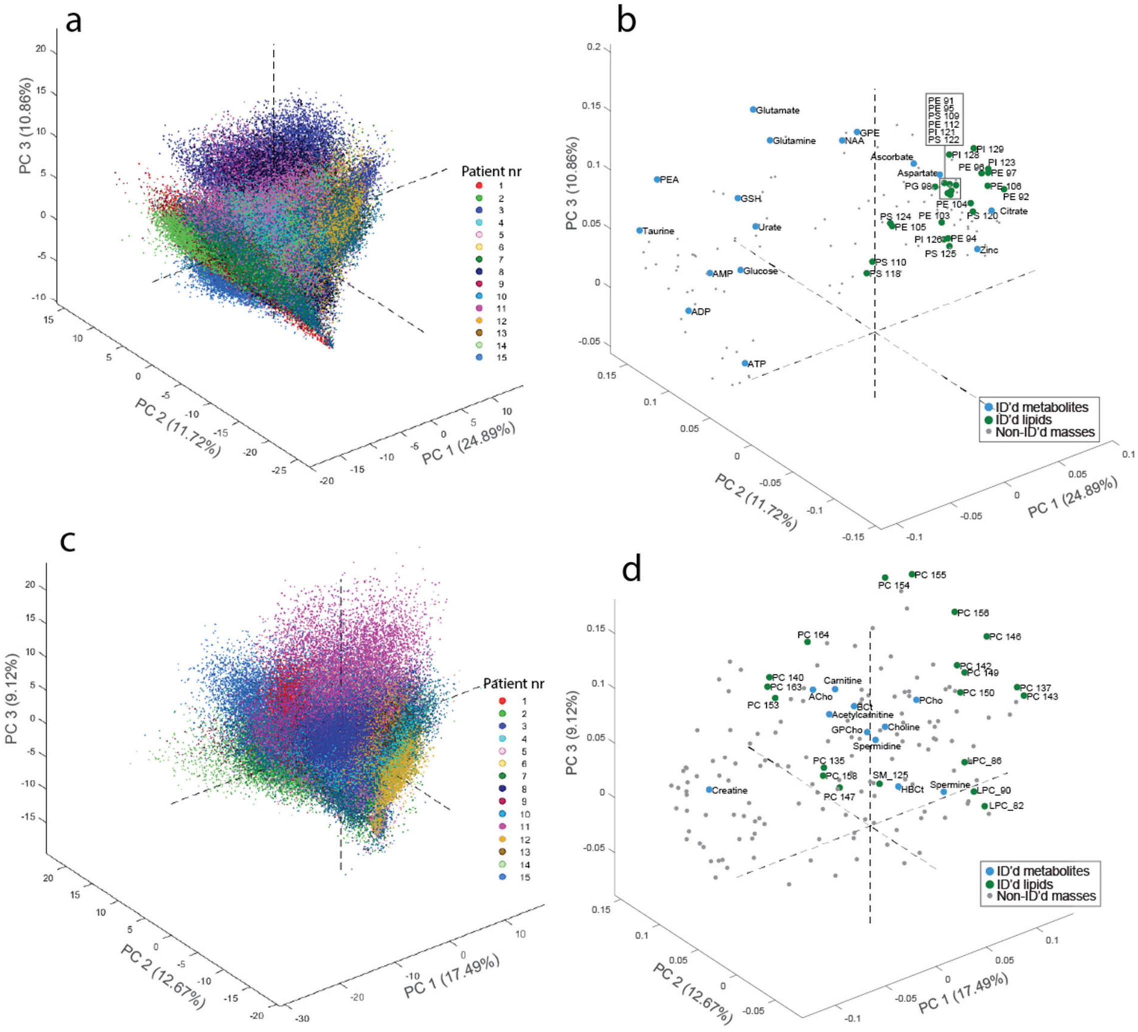

Supplementary Figure S1: Principal component analysis (PCA) of metabolites and lipids detected with MALDI MSI. The three first principal components (PC) are shown with (a) scores plot and (b) loadings plot for negative ion mode dataset, and (c) scores plot and (d) loadings plot for positive ion mode dataset. Data points in the scores plots (a, c) represent single mass spectra and are colored by patient origin. In the (b, d) loadings plots (b, d), identified metabolites and lipids are shown as blue and green circles, respectively. ACho = acetylcholine, ADP = adenosine diphosphate, AMP = adenosine monophosphate, ATP = adenosine triphosphate, BCt = butylyl-L-carnitine, GPE = glycerophoshpoethanolamine, HBCt = hydroxybutyrylcarnitine, NAA = *N*-acetylaspartate, LPC = lysophosphatidylcholine, PCho=phosphocholine, PE= phosphoethanolamines, PE=phosphatidylethanolamines, PC=phosphatidylcholines, PG=phosphatidylglycerols, PI=phosphatidylinositols, PS=phosphatidylserine and SM=sphingomyelin. The particular phospholipids and their chain-composition can be identified by the number and found in Supplementary Table S4.

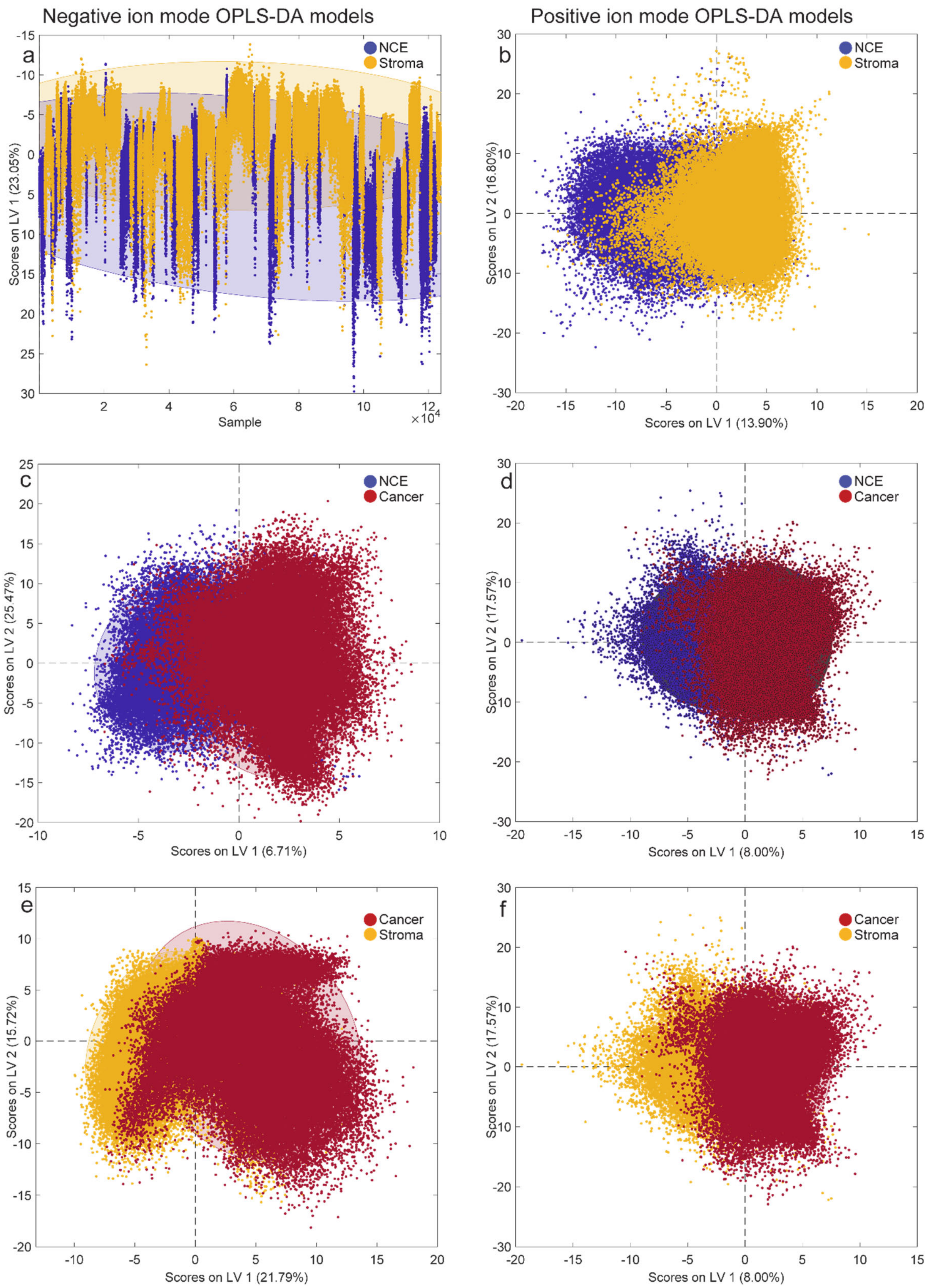

Supplementary Figure S2: Score plots for orthogonalized partial least squares discrimination analysis (OPLS-DA) models. The models are pairwise comparing (a,b) non-cancer epithelium (NCE) to stroma, (c,d) NCE to cancer and (e,f) cancer to stroma. The variation explained by the tissue types are separated along the first latent variable (LV). For the model in (a), only one LV was extracted and the scores plot therefore have the LV on the y-axis and the order of the spectra on the X-axis. Additionally, for visual purposes and to better interpret the scores plot together with the corresponding loadings plot (Figure 1), the scores in panel a) have been reversed.

Supplementary Table S5: Full peak list for masses in negative ion mode with NEDC matrix. Table includes mean peak height and standard deviation (SD) across different tissue types. Non-cancer epithelium (NCE), stroma and cancer were pairwise compared and here we present $\log_2$ fold change (Log_2FC), adjusted p-values from linear mixed models and variable importance on the projection (VIP) scores from orthogonal projections to latent structures discriminant Analysis (OPLS-DA) models. PE = phosphoethanolamines, PC = phosphatidylcholines, PG = phosphatidylglycerols, PI = phosphatidylinositols and PS = phosphatidylserine.

Mass, *m/z*	ID	ID in OPLS-DA	Mean NCE (SD)	Mean cancer (SD)	Mean stroma (SD)	NCE vs stroma			Cancer vs NCE			Cancer vs stroma		
						Log_2FC	p-value	VIP	Log_2FC	p-value	VIP	Log_2FC	p-value	VIP
87.056			0.41 (0.29)	0.32 (0.26)	0.26 (0.2)	0.657	3.80E-05	0.99	-0.358	0.39	0.9	0.3	0.031	0.42
124.02	Taurine	Taurine	21.61 (10.74)	21.65 (10.75)	30.91 (12.91)	-0.516	1.60E-09	1.11	0.003	0.15	0.01	-0.514	0.078	1.09
124.34			0.29 (0.17)	0.31 (0.21)	0.49 (0.27)	-0.757	2.50E-10	1.21	0.096	0.095	0.34	-0.661	0.13	1.04
129.04			0.87 (0.76)	0.48 (0.35)	0.53 (0.43)	0.715	5.10E-07	0.9	-0.858	0.0033	1.8	-0.143	0.41	0.18
130.06			0.53 (0.28)	0.39 (0.22)	0.5 (0.27)	0.084	0.48	0.18	-0.443	0.062	1.47	-0.358	0.33	0.65
132.03	Aspartate	Aspartate	5.96 (3.47)	3.99 (2.03)	3.8 (2.13)	0.649	4.00E-08	1.2	-0.579	2.50E-06	1.88	0.07	0.45	0.14
135.05			0.6 (0.31)	0.91 (0.56)	0.61 (0.33)	-0.024	0.32	0.02	0.601	0.001	1.67	0.577	0.022	0.99
140.02	Phosphoethanolamine	PEA	3.38 (2.08)	4.23 (2.34)	4.47 (2.25)	-0.403	1.00E-06	0.75	0.324	0.15	1	-0.08	0.53	0.16
145.07	Glutamine	Glutamine	2.7 (1.13)	2.91 (1.96)	2.8 (1.18)	-0.052	0.084	0.14	0.108	0.64	0.34	0.056	0.6	0.11
146.06	Glutamate	Glutamate	17.88 (6.97)	18.86 (7.91)	17.37 (7)	0.042	0.29	0.11	0.077	0.62	0.35	0.119	0.46	0.3
147.04			2.42 (1.07)	1.78 (0.79)	1.71 (0.76)	0.501	3.20E-07	1.2	-0.443	8.90E-05	1.82	0.058	0.37	0.14
166.03			1.73 (0.72)	1.68 (0.76)	2.25 (0.97)	-0.379	3.30E-05	0.87	-0.042	0.42	0.19	-0.421	0.2	0.93
167.03	Urate	Urate	2.47 (0.81)	3.02 (1.27)	3.11 (1.27)	-0.332	0.00018	0.84	0.29	0.0022	1.3	-0.042	0.33	0.11
171.01			2.01 (0.95)	3.06 (1.87)	2.26 (0.94)	-0.169	0.0049	0.41	0.606	0.028	1.69	0.437	0.25	0.82
173.01			2.61 (3.1)	1.84 (1.48)	1.82 (1.8)	0.52	0.00016	0.53	-0.504	0.22	0.93	0.016	0.18	0.02
174.04	*N*-acetylaspartate	NAA	2.58 (1.9)	4.29 (3.17)	2.18 (1.09)	0.243	0.51	0.44	0.734	0.22	1.59	0.977	0.0012	1.29
174.83	Zinc	Zinc	6.31 (4.98)	3.04 (2.69)	3.64 (3.1)	0.794	1.10E-07	1.04	-1.054	0.00019	2.19	-0.26	0.27	0.31
175.02	Ascorbate	Ascorbate	2.43 (1.64)	2 (1.15)	1.62 (1.09)	0.585	1.60E-05	0.93	-0.281	0.01	0.84	0.304	0.077	0.5
175.99			4.21 (3.32)	5 (3.15)	6.28 (3.23)	-0.577	7.50E-09	0.95	0.248	0.52	0.66	-0.329	0.045	0.59
176.54			0.44 (0.34)	0.24 (0.16)	0.26 (0.2)	0.759	1.00E-06	1.02	-0.874	0.0013	2.05	-0.115	0.47	0.22
177.98			1.46 (1.2)	1.75 (1.16)	1.99 (1.16)	-0.447	5.60E-07	0.68	0.261	0.26	0.66	-0.185	0.2	0.31
180.04			1.1 (0.49)	1.41 (0.7)	0.82 (0.37)	0.424	3.10E-06	1.01	0.358	0.081	1.29	0.782	6.40E-10	1.46
181.03			3.61 (1.55)	3.84 (2.26)	4 (1.55)	-0.148	0.00083	0.38	0.089	0.52	0.3	-0.059	0.18	0.13
181.96			6.91 (3.4)	7.2 (3.75)	10.12 (4.35)	-0.55	2.00E-08	1.14	0.059	0.058	0.22	-0.491	0.2	1.01
182.83			3.24 (1.22)	3.39 (1.23)	3.32 (1.42)	-0.035	0.52	0.1	0.065	0.62	0.34	0.03	0.35	0.08
184.99			0.73 (0.42)	1.06 (0.98)	1.08 (0.59)	-0.565	9.80E-05	0.93	0.538	4.00E-04	1.05	-0.027	0.43	0.03
191.02	Citrate	Citrate	115.78 (69.1)	62.38 (42.2)	66.45 (51.28)	0.801	1.50E-12	1.24	-0.892	2.30E-07	2.41	-0.091	0.48	0.13
191.12			6.46 (5.21)	2.64 (2.46)	3.17 (3.26)	1.027	2.10E-12	1.2	-1.291	1.00E-05	2.47	-0.264	0.16	0.27
191.4			2.78 (2.5)	1.13 (1.07)	1.26 (1.38)	1.142	1.20E-11	1.22	-1.299	5.00E-05	2.31	-0.157	0.12	0.15
197.94			6.61 (3.97)	6.31 (3.44)	9.91 (4.34)	-0.584	4.80E-10	1.13	-0.067	0.56	0.22	-0.651	0.0012	1.24
208.98			1.12 (0.59)	1.71 (1.14)	1.15 (0.73)	-0.038	0.077	0.07	0.61	0.4	1.56	0.572	0.24	0.86
213.02			2.86 (1.11)	3.14 (1.84)	3.67 (1.38)	-0.36	6.20E-07	0.93	0.135	0.68	0.46	-0.225	0.0066	0.5

213.94			0.47 (0.33)	0.61 (0.37)	0.65 (0.34)	-0.468	1.10E-05	0.82	0.376	0.1	1.05	-0.092	0.57	0.19
214.05	Glycerylphosphoryl-ethanolamine	GPE	6.93 (5.73)	12.22 (13.94)	4.07 (5.73)	0.768	6.40E-05	0.75	0.818	0.062	1.2	1.586	0.01	1.13
214.93			1.08 (0.42)	1.12 (0.54)	1.5 (0.56)	-0.474	3.60E-09	1.16	0.052	0.51	0.23	-0.421	0.0037	0.97
215.03	Glucose	Glucose	95.23 (38.44)	81.35 (45.8)	117.73 (46.8)	-0.306	0.0016	0.76	-0.227	0.42	0.86	-0.533	0.024	1.1
215.16			2.4 (1.07)	2.36 (1.22)	3.57 (1.36)	-0.573	3.70E-10	1.29	-0.024	0.63	0.09	-0.597	3.00E-05	1.27
215.44			0.99 (0.48)	0.97 (0.55)	1.59 (0.65)	-0.684	6.70E-16	1.38	-0.029	0.51	0.12	-0.713	4.20E-05	1.36
216.31			1.04 (0.43)	1.11 (0.54)	1.56 (0.6)	-0.585	2.10E-12	1.33	0.094	0.18	0.39	-0.491	0.0011	1.1
217.93			0.53 (0.4)	0.6 (0.46)	0.93 (0.58)	-0.811	6.40E-08	1.09	0.179	0.13	0.38	-0.632	0.14	0.9
218.31			0.33 (0.15)	0.3 (0.15)	0.44 (0.17)	-0.415	6.70E-09	0.97	-0.138	0.25	0.53	-0.553	3.70E-10	1.17
226.98			1.95 (1.07)	2.98 (2.25)	2.05 (1.32)	-0.072	0.023	0.12	0.612	0.63	1.41	0.54	0.5	0.77
239.92			1.42 (0.69)	1.41 (0.76)	2.05 (0.96)	-0.53	2.30E-06	1.05	-0.01	0.42	0.02	-0.54	0.0085	1.03
241.92			0.97 (0.52)	0.91 (0.42)	1.46 (0.72)	-0.59	6.40E-06	1.08	-0.092	0.36	0.33	-0.682	0.03	1.23
246.77			2.35 (1.77)	1.34 (0.73)	1.53 (0.95)	0.619	2.30E-05	0.97	-0.81	3.10E-05	2.07	-0.191	0.51	0.32
248.99			5.54 (5.43)	2.45 (1.9)	3.14 (2.23)	0.819	0.00044	1	-1.177	3.50E-05	2.12	-0.358	0.42	0.49
255.89			1.96 (0.95)	2.05 (1.09)	2.93 (1.4)	-0.58	3.20E-07	1.11	0.065	0.3	0.25	-0.515	0.17	0.98
264.94			3.24 (2.95)	1.51 (1.28)	1.86 (1.77)	0.801	3.50E-07	0.93	-1.101	0.00019	2.09	-0.301	0.41	0.33
267.07			5.24 (3.49)	5.2 (3.69)	4.82 (3.67)	0.121	0.43	0.18	-0.011	0.48	0.03	0.109	0.25	0.16
270.99			2.13 (1.83)	1.92 (2.08)	3.44 (2.79)	-0.692	4.40E-05	0.78	-0.15	0.59	0.28	-0.841	0.14	0.87
271.86			0.99 (0.63)	0.98 (0.61)	1.4 (0.74)	-0.5	1.50E-05	0.88	-0.015	0.5	0.03	-0.515	0.34	0.89
273.86			0.82 (0.49)	0.76 (0.44)	1.13 (0.51)	-0.463	0.00019	0.91	-0.11	0.54	0.35	-0.572	0.062	1.08
274.09			1.42 (0.76)	1.53 (1)	1.78 (0.96)	-0.326	0.0027	0.6	0.108	0.6	0.33	-0.218	0.27	0.38
275.09			0.72 (0.53)	1.01 (0.78)	0.9 (0.63)	-0.322	0.064	0.45	0.488	0.0076	1.09	0.166	0.058	0.24
279.03			3.3 (2.22)	3.87 (2.58)	3.12 (2.04)	0.081	0.29	0.13	0.23	0.21	0.62	0.311	0.22	0.49
286.97			2.35 (1.41)	1.77 (1.47)	3.23 (2.09)	-0.459	0.001	0.7	-0.409	0.0092	1.05	-0.868	0.052	1.11
288.03			1.45 (1.19)	1.1 (0.9)	1.03 (0.89)	0.493	0.0018	0.64	-0.399	0.011	0.92	0.095	0.52	0.12
289.03			5.81 (4.23)	3.77 (2.72)	3.81 (3.58)	0.609	1.20E-05	0.79	-0.624	0.0076	1.57	-0.015	0.45	0.02
290.07			1.07 (0.65)	0.95 (0.6)	0.87 (0.47)	0.299	0.15	0.57	-0.172	0.4	0.53	0.127	0.045	0.22
291.06			1.28 (0.65)	1.27 (0.92)	1.33 (0.74)	-0.055	0.1	0.09	-0.011	0.46	0.03	-0.067	0.1	0.1
292.06			1.42 (1.01)	2.23 (2.1)	1.12 (0.85)	0.342	0.44	0.51	0.651	0.56	1.2	0.994	0.014	1.04
302.06			3.58 (2.99)	3.03 (2.72)	2.02 (2.35)	0.826	0.00043	0.91	-0.241	0.0034	0.53	0.585	0.31	0.59
303.04			2.1 (1.25)	2.51 (1.55)	2.89 (1.99)	-0.461	0.00088	0.66	0.257	0.003	0.76	-0.203	0.6	0.31
304.05			2.29 (1.28)	1.92 (1.12)	2.12 (1.25)	0.111	0.48	0.21	-0.254	0.03	0.84	-0.143	0.29	0.25
305.05			1.75 (0.81)	1.92 (0.89)	2.3 (1.06)	-0.394	3.10E-07	0.84	0.134	0.039	0.53	-0.261	0.13	0.57
306.068	Glutathione	GSH	28.23 (14.54)	27.35 (20.59)	29.75 (17.63)	-0.076	0.0061	0.14	-0.046	0.31	0.13	-0.121	0.17	0.19
313.85			0.9 (0.52)	1.02 (0.55)	1.41 (0.72)	-0.648	7.10E-09	1.1	0.181	0.15	0.59	-0.467	0.021	0.85
315.84			1.05 (0.58)	0.97 (0.48)	1.41 (0.66)	-0.425	6.00E-04	0.84	-0.114	0.55	0.43	-0.54	0.0083	1.05
323.02			1.06 (0.89)	1.19 (0.78)	1.23 (0.88)	-0.215	0.032	0.31	0.167	0.31	0.44	-0.048	0.34	0.07
328.06			0.9 (0.47)	0.94 (0.61)	0.99 (0.53)	-0.138	0.031	0.27	0.063	0.62	0.2	-0.075	0.14	0.13
329.81			0.73 (0.39)	0.78 (0.43)	1.05 (0.52)	-0.524	1.10E-06	0.99	0.096	0.26	0.31	-0.429	0.039	0.83
331.82			0.95 (0.55)	0.85 (0.42)	1.27 (0.6)	-0.419	0.00054	0.83	-0.16	0.59	0.56	-0.579	0.0013	1.12

338.98			0.81 (0.58)	0.9 (0.63)	1.43 (0.97)	-0.82	2.40E-08	1.05	0.152	0.00017	0.41	-0.668	0.055	0.9
344.03			0.74 (0.4)	0.75 (0.51)	0.79 (0.46)	-0.094	0.045	0.16	0.019	0.55	0.05	-0.075	0.2	0.12
346.05	Adenosine monophosphate	AMP	4.47 (5.07)	5.57 (4.19)	7.45 (7.17)	-0.737	5.00E-05	0.68	0.317	0.39	0.65	-0.42	0.065	0.46
389.78			0.87 (0.57)	0.85 (0.47)	1.12 (0.62)	-0.364	0.0074	0.62	-0.034	0.38	0.12	-0.398	0.016	0.7
402.98			0.95 (0.6)	1.39 (0.93)	1.18 (0.81)	-0.313	0.0013	0.46	0.549	0.0017	1.37	0.236	0.095	0.35
426.01	Adenosine diphosphate	ADP	2.89 (2.77)	3.78 (2.78)	5.96 (5.02)	-1.044	3.40E-10	1.02	0.387	0.025	0.86	-0.657	0.018	0.76
467.08			0.62 (0.53)	0.64 (0.59)	0.54 (0.44)	0.199	0.21	0.28	0.046	0.34	0.07	0.245	0.028	0.3
482.93			0.52 (0.89)	0.49 (0.69)	1.28 (1.56)	-1.3	1.40E-09	0.81	-0.086	0.56	0.11	-1.385	6.50E-06	0.89
490.17			0.8 (0.42)	0.84 (0.56)	1.07 (0.58)	-0.42	2.10E-05	0.77	0.07	0.45	0.25	-0.349	0.02	0.59
505.97	Adenosine triphosphate	ATP	2.18 (3.97)	1.82 (2.82)	5.17 (6.79)	-1.246	2.40E-06	0.74	-0.26	0.28	0.3	-1.506	0.00037	0.89
521.95			0.47 (0.45)	0.48 (0.37)	1 (0.95)	-1.089	2.90E-11	0.95	0.03	0.4	0.1	-1.059	4.80E-06	0.98
527.94			0.56 (0.64)	0.49 (0.49)	1.25 (1.37)	-1.158	3.80E-08	0.86	-0.193	0.56	0.34	-1.351	0.00014	1
543.92			0.5 (0.57)	0.44 (0.39)	0.97 (1.01)	-0.956	8.70E-07	0.79	-0.184	0.49	0.36	-1.14	3.00E-04	0.94
606.05			1.74 (1.36)	2.62 (1.84)	1.13 (0.68)	0.623	0.014	0.96	0.59	0.12	1.37	1.213	1.00E-09	1.5
611.12			0.66 (0.44)	0.72 (0.57)	1.05 (0.65)	-0.67	6.90E-08	0.97	0.126	0.48	0.32	-0.544	0.021	0.77
625.19			0.61 (0.54)	1.21 (0.96)	0.42 (0.29)	0.538	0.019	0.77	0.988	5.50E-05	1.82	1.527	1.10E-14	1.54
658.83			1.3 (1.26)	1.06 (0.67)	1.07 (0.87)	0.281	0.46	0.35	-0.294	0.21	0.68	-0.014	0.52	0.02
687.52			2.19 (1.57)	2.63 (2.03)	1.83 (1.24)	0.259	0.0048	0.41	0.264	0.021	0.63	0.523	0.00032	0.72
714.48	PE (16:0_18:2)	PE 91	2.37 (2.43)	3.69 (3.54)	0.69 (0.83)	1.78	1.40E-27	1.53	0.639	0.5	1.09	2.419	1.10E-18	1.6
716.48	PE (16:0_18:1)	PE 92	9.25 (7.67)	10.97 (8.95)	1.98 (3.17)	2.224	1.70E-35	1.87	0.246	0.4	0.54	2.47	6.70E-23	1.75
722.49			1.4 (1.37)	1.04 (0.85)	0.81 (0.7)	0.789	0.00035	0.92	-0.429	0.042	0.89	0.361	0.14	0.44
738.49	PE (16:0_20:4)	PE 94	1.99 (1.36)	2.49 (2.01)	1.1 (0.76)	0.855	7.40E-10	1.3	0.323	0.62	0.73	1.179	1.30E-07	1.31
740.49	PE (36:3)	PE 95	3.18 (3.07)	4.69 (4.35)	0.86 (1.02)	1.887	1.50E-24	1.65	0.561	0.48	1.02	2.447	5.90E-17	1.65
742.51	PE (18:1_18:1)	PE 96	10.17 (8.65)	13.65 (11.3)	2.44 (3.24)	2.059	8.30E-30	1.83	0.425	0.59	0.89	2.484	3.60E-22	1.77
744.52	PE (18:0_18:1)	PE 97	8.85 (5.93)	12.44 (8.71)	3.04 (3.13)	1.542	6.20E-25	1.81	0.491	0.37	1.21	2.033	8.50E-17	1.84
747.48	PG (16:0_18:1)	PG 98	1.28 (0.74)	1.73 (1.04)	0.87 (0.52)	0.557	3.70E-05	1	0.435	0.18	1.27	0.992	3.50E-07	1.45
748.49			1.37 (0.93)	1.57 (0.88)	0.93 (0.64)	0.559	9.80E-05	0.89	0.197	0.6	0.6	0.755	4.70E-05	1.19
750.52			1.82 (1.41)	2.09 (1.11)	1.46 (1.13)	0.318	0.047	0.45	0.2	0.44	0.6	0.518	0.00069	0.82
752.52			1.19 (0.6)	1.54 (0.71)	0.81 (0.43)	0.555	1.40E-08	1.12	0.372	0.15	1.37	0.927	2.10E-10	1.64
760.48			0.86 (0.54)	1.06 (0.64)	0.98 (0.54)	-0.188	0.12	0.34	0.302	0.27	0.89	0.113	0.59	0.21
762.48	PE (16:0_22:6)	PE 103	1.73 (1.34)	2.27 (1.92)	0.95 (0.7)	0.865	1.80E-09	1.19	0.392	0.26	0.84	1.257	5.70E-08	1.32
764.49	PE (18:1_20:4)	PE 104	2.64 (1.74)	2.85 (1.81)	1.13 (0.93)	1.224	1.50E-18	1.65	0.11	0.44	0.32	1.335	4.10E-10	1.6
766.5	PE (18:0_20:4)	PE 105	8.84 (5.48)	9.96 (5.75)	6.48 (4.3)	0.448	0.00012	0.76	0.172	0.49	0.53	0.62	0.00022	1
770.52	PE (18:1_20:1)	PE 106	2 (1.43)	2.11 (1.52)	0.78 (0.64)	1.358	1.30E-23	1.71	0.077	0.51	0.2	1.436	2.00E-14	1.56
773.52			0.86 (0.6)	1.07 (0.84)	0.51 (0.29)	0.754	6.40E-09	1.23	0.315	0.58	0.73	1.069	1.50E-07	1.29
774.49			1.08 (0.57)	1.31 (0.67)	0.8 (0.44)	0.433	0.00022	0.87	0.279	0.52	0.96	0.711	1.20E-05	1.27
786.48	PS (36:3)	PS 109	2.14 (1.39)	2.37 (1.71)	0.99 (0.74)	1.112	5.40E-16	1.59	0.147	0.43	0.4	1.259	4.20E-08	1.47
788.5	PS (18:0_18:1)	PS 110	12.41 (7.27)	13.3 (7.61)	10.54 (6.42)	0.236	0.0065	0.43	0.1	0.39	0.32	0.336	0.1	0.59
790.51			3.79 (2.52)	5.94 (4.8)	2.29 (1.66)	0.727	4.70E-08	1.12	0.648	0.026	1.37	1.375	1.90E-10	1.44
792.53	PE (20:1_20:4)	PE 112	1.82 (1.11)	2.74 (1.89)	1.08 (0.7)	0.753	9.70E-08	1.25	0.59	0.041	1.45	1.343	1.60E-12	1.59

794.52			1.95 (1.09)	2.68 (1.42)	1.39 (0.8)	0.488	7.80E-06	0.92	0.459	0.027	1.44	0.947	1.10E-10	1.51
800.48			0.76 (0.43)	1 (0.62)	0.49 (0.22)	0.633	6.30E-09	1.3	0.396	0.42	1.11	1.029	2.50E-09	1.51
802.49			0.94 (0.47)	1.28 (0.68)	0.64 (0.31)	0.555	6.30E-09	1.18	0.445	0.079	1.43	1	1.20E-10	1.62
809.49			1.16 (1.04)	1.33 (1.37)	0.45 (0.41)	1.366	4.60E-23	1.48	0.197	0.48	0.35	1.563	1.70E-12	1.27
810.49			1.88 (1.16)	1.86 (1.07)	1.78 (1.1)	0.079	0.17	0.14	-0.015	0.15	0.04	0.063	0.12	0.11
812.52	PS (18:0_20:3)	PS 118	2.39 (1.52)	2.12 (1.17)	1.98 (1.29)	0.272	0.0079	0.47	-0.173	0.55	0.56	0.099	0.1	0.17
814.5			1.49 (0.9)	1.23 (0.6)	0.82 (0.5)	0.862	2.60E-12	1.44	-0.277	0.16	0.93	0.585	4.40E-05	1.07
816.51	PS (38:1)	PS 120	1.58 (0.93)	1.66 (1.04)	0.94 (0.59)	0.749	1.40E-09	1.28	0.071	0.46	0.22	0.82	1.20E-06	1.22
833.5	PI (16:0_18:2)	PI 121	1.19 (0.86)	1.81 (1.54)	0.54 (0.42)	1.14	7.20E-18	1.52	0.605	0.39	1.23	1.745	8.50E-14	1.56
834.51	PS (18:0_22:6)	PS 122	3.03 (1.99)	3.48 (2.15)	1.59 (1.14)	0.93	9.80E-14	1.39	0.2	0.28	0.58	1.13	6.70E-12	1.5
835.49	PI (16:0_18:1)	PI 123	5.08 (3.85)	5.84 (4.71)	1.55 (1.74)	1.713	5.60E-29	1.8	0.201	0.32	0.46	1.914	3.90E-18	1.64
838.51	PS (40:4)	PS 124	1.46 (0.86)	1.83 (1.21)	1.24 (0.74)	0.236	0.01	0.44	0.326	0.0088	0.89	0.562	8.20E-05	0.88
844.55	PS (18:1_22:0)	PS 125	1.06 (0.6)	1.17 (0.75)	0.77 (0.46)	0.461	0.00015	0.86	0.142	0.11	0.4	0.604	2.20E-05	0.95
857.47	PI (16:1_20:4)	PI 126	2.22 (1.74)	2.75 (2.06)	1.28 (0.94)	0.794	1.40E-10	1.11	0.309	0.18	0.73	1.103	1.70E-10	1.32
859.49			1.74 (1.93)	2 (1.39)	0.79 (0.66)	1.139	9.70E-14	1.14	0.201	0.5	0.44	1.34	4.60E-12	1.53
861.5	PI (18:1_18:1)	PI 128	3.65 (2.9)	5.89 (5.2)	1.16 (1.34)	1.654	1.10E-22	1.71	0.69	0.28	1.31	2.344	1.40E-20	1.68
863.53	PI (18:0_18:1)	PI 129	3.47 (2.76)	4.79 (4.41)	0.92 (1.2)	1.915	1.00E-30	1.82	0.465	0.36	0.9	2.38	3.70E-22	1.63
881.48			1.01 (0.84)	1.4 (1.41)	0.49 (0.34)	1.044	6.10E-13	1.35	0.471	0.22	0.85	1.515	6.80E-12	1.29
883.49			2.15 (1.37)	2.37 (1.43)	1.36 (0.89)	0.661	3.20E-09	1.1	0.141	0.48	0.41	0.801	1.40E-07	1.22
885.51			16.49 (10.51)	19.93 (10.57)	15.26 (9.84)	0.112	0.11	0.19	0.273	0.00092	0.87	0.385	4.30E-05	0.68
889.55			2.31 (2.46)	2.78 (2.41)	0.77 (0.71)	1.585	6.10E-18	1.44	0.267	0.5	0.52	1.852	1.70E-17	1.56
909.52			1.64 (1.49)	2.49 (2.58)	0.66 (0.67)	1.313	1.00E-17	1.38	0.602	0.066	1.01	1.916	7.70E-19	1.39
911.53			1.35 (0.98)	1.98 (1.63)	0.69 (0.53)	0.968	8.10E-12	1.35	0.553	0.018	1.16	1.521	5.60E-15	1.49
913.54			1.08 (0.82)	1.81 (1.59)	0.66 (0.45)	0.71	1.00E-06	1.05	0.745	0.0046	1.39	1.455	3.70E-12	1.41

Supplementary Table S6: Full peak list for masses in positive ion mode with DHB matrix. Table includes mean peak height and standard deviation (SD) across different tissue types. Non-cancer epithelium (NCE), stroma and cancer were pairwise compared and here we present log_2 fold change (Log_2FC), adjusted p-values from linear mixed models and variable importance on the projection (VIP) scores from orthogonal projections to latent structures discriminant Analysis (OPLS-DA) models. LPC = lysophosocholines, PC = phosphatidylcholines and SM = sphingomyelin.

Mass *m/z*	ID	ID in OPLS-DA	Mean NCE (SD)	Mean cancer (SD)	Mean stroma (SD)	NCE vs stroma			Cancer vs NCE			Cancer vs stroma		
						Log_2FC	p-vale	VIP	Log_2FC	p-value	VIP	Log_2FC	p-value	VIP
100.18			2.57 (3.19)	4.26 (3.8)	1.94 (2.13)	0.406	0.12	0.49	0.729	0.035	1.19	1.135	0.0033	1.4
104.17	Choline	Choline	206.41 (124.49)	355.74 (198.58)	183.96 (122.12)	0.166	0.29	0.36	0.785	0.00096	2.04	0.951	0.0025	1.82
123.11			2.08 (1.69)	2.24 (1.55)	1.98 (1.42)	0.071	0.54	0.13	0.107	0.31	0.25	0.178	0.38	0.34
132.13	Creatine	Creatine	26.7 (16.68)	24.04 (16.4)	36 (21.38)	-0.431	0.00014	0.9	-0.151	0.08	0.41	-0.583	0.15	1.15
136.11			8.23 (6.01)	7.76 (6.38)	10.01 (6.7)	-0.282	0.0074	0.53	-0.085	0.44	0.2	-0.367	0.018	0.65
142.08			0.78 (0.44)	1.05 (0.58)	1.12 (0.63)	-0.522	1.70E-05	1.14	0.429	0.011	1.27	-0.093	0.12	0.24
146.165	Acetylcholine	ACho	0.91 (0.54)	1.32 (0.76)	1 (0.58)	-0.136	0.2	0.3	0.537	0.00051	1.48	0.401	0.06	0.91
146.215	Spermidine	Spermidine	1.17 (1.2)	1.4 (1.53)	0.75 (0.65)	0.642	0.00036	0.91	0.259	0.45	0.42	0.9	1.50E-06	1.07
147.02			8.85 (5.45)	7.45 (5.83)	6.27 (4.3)	0.497	0.00019	1.04	-0.248	0.35	0.63	0.249	0.07	0.45
147.13			4.02 (1.92)	5.58 (3.95)	4.21 (1.86)	-0.067	0.31	0.2	0.473	0.0062	1.18	0.406	0.17	0.87
148.11			2.35 (1.56)	3.83 (2.6)	2.7 (1.64)	-0.2	0.25	0.43	0.705	0.0012	1.61	0.504	0.047	1
152.1			2.76 (2.17)	3.47 (3.24)	2.91 (2.21)	-0.076	0.21	0.14	0.33	0.062	0.63	0.254	0.55	0.39
160.18			3.53 (2.15)	5.56 (2.74)	3.76 (2.47)	-0.091	0.043	0.19	0.655	1.70E-05	1.92	0.564	0.064	1.27
162.16	Carnitine	Carnitine	19.21 (11.43)	32.24 (18.53)	21.03 (14.19)	-0.131	0.01	0.27	0.747	2.30E-09	1.93	0.616	0.058	1.26
163			3.74 (2.26)	3.41 (2.41)	2.9 (1.87)	0.367	0.0078	0.8	-0.133	0.25	0.36	0.234	0.29	0.46
164.05			0.89 (0.33)	0.97 (0.33)	1.07 (0.4)	-0.266	0.0028	0.91	0.124	0.075	0.56	-0.142	0.29	0.55
165.11			0.54 (0.39)	0.92 (0.65)	0.77 (0.52)	-0.512	1.90E-05	0.95	0.769	0.0063	1.67	0.257	0.55	0.5
166.1			1.42 (0.73)	1.36 (0.89)	1.15 (0.59)	0.304	0.0055	0.8	-0.062	0.48	0.19	0.242	0.11	0.52
170.02			2.84 (1.99)	2.37 (1.41)	4.43 (2.25)	-0.641	6.70E-06	1.37	-0.261	0.021	0.72	-0.902	0.017	1.83
174.94			2.4 (1.76)	3.18 (1.97)	2.81 (2.04)	-0.228	0.074	0.42	0.406	0.0042	1.05	0.178	0.53	0.36
180.03			0.63 (0.36)	0.87 (0.49)	0.84 (0.49)	-0.415	0.00051	0.88	0.466	0.046	1.34	0.051	0.23	0.13
180.94			0.97 (0.5)	1.09 (0.53)	1.1 (0.59)	-0.181	0.035	0.45	0.168	0.0011	0.56	-0.013	0.55	0.05
184.11	Phosphocholine	PCho	149.78 (68.76)	122.91 (89.92)	123.26 (58.56)	0.281	6.00E-04	0.82	-0.285	0.052	0.83	-0.004	0.37	0.01
184.49			4.65 (2.22)	3.82 (3.05)	3.77 (1.85)	0.303	0.00056	0.85	-0.284	0.061	0.76	0.019	0.33	0.04
185.3			2.98 (1.44)	2.47 (1.95)	2.46 (1.24)	0.277	0.0014	0.76	-0.271	0.089	0.72	0.006	0.35	0.01
186			7.49 (4.71)	7.87 (4.43)	12.51 (6.72)	-0.74	4.60E-10	1.51	0.071	0.00045	0.21	-0.669	0.016	1.44
186.12			2.62 (1.08)	2.24 (1.35)	2.27 (1.03)	0.207	0.026	0.65	-0.226	0.28	0.77	-0.019	0.45	0.05
188			0.89 (0.49)	0.92 (0.47)	1.39 (0.73)	-0.643	1.40E-08	1.41	0.048	0.0012	0.16	-0.595	0.033	1.36
189.2			0.48 (0.3)	1.14 (1.05)	0.38 (0.25)	0.337	0.27	0.68	1.248	0.00024	1.88	1.585	2.70E-05	1.78
190.06			0.47 (0.28)	0.73 (0.67)	0.42 (0.26)	0.162	0.49	0.39	0.635	0.013	1.18	0.798	0.2	1.19
190.96			2.33 (1.88)	3.06 (2.2)	2.9 (2.25)	-0.316	0.0072	0.53	0.393	0.059	0.89	0.077	0.3	0.14
196.92			0.98 (0.62)	1.29 (0.71)	1.18 (0.76)	-0.268	0.02	0.54	0.397	0.00093	1.16	0.129	0.55	0.3
201.98			6.36 (5.67)	7.72 (5.83)	11.78 (8.87)	-0.889	1.20E-07	1.28	0.28	0.0081	0.6	-0.61	0.014	0.99

203.1			2.13 (1.12)	2.26 (1.2)	2.88 (1.44)	-0.435	5.90E-05	1.07	0.085	0.0099	0.27	-0.35	0.18	0.88
203.27	Spermine	Spermine	21.42 (20.34)	13.58 (13.38)	8.47 (9.52)	1.339	1.30E-10	1.65	-0.657	0.061	1.2	0.681	3.10E-05	0.85
203.97			1.21 (0.98)	1.45 (1.05)	2.12 (1.58)	-0.809	1.00E-07	1.22	0.261	0.011	0.62	-0.548	0.028	0.91
204.17	Acetylcarnitine	Acetylcarnitine	15.78 (9.2)	27.26 (15.12)	17.72 (12.81)	-0.167	0.013	0.33	0.789	0.00017	2.07	0.621	0.076	1.26
206.1			8.16 (4.36)	8.33 (5.28)	9.81 (4.56)	-0.266	0.027	0.72	0.03	0.42	0.09	-0.236	0.15	0.58
208.04			0.68 (0.22)	0.76 (0.26)	0.82 (0.3)	-0.27	2.20E-05	0.93	0.16	0.0038	0.85	-0.11	0.22	0.36
217.98			0.83 (0.61)	1.2 (0.83)	1.4 (0.99)	-0.754	3.30E-08	1.21	0.532	0.018	1.22	-0.222	0.0048	0.41
222.07			7.56 (5.05)	10.19 (8.47)	11.14 (7)	-0.559	9.40E-06	1.06	0.431	0.14	0.91	-0.129	0.034	0.24
224.07			0.8 (0.37)	0.96 (0.6)	1.03 (0.49)	-0.365	0.00041	0.97	0.263	0.31	0.76	-0.102	0.056	0.25
225.25			1.95 (1.79)	1.21 (1.01)	0.89 (0.88)	1.132	7.40E-10	1.54	-0.688	0.042	1.34	0.443	5.40E-05	0.66
232.2	Butyryl-L-carnitine	BCt	2.03 (1.25)	2.37 (1.22)	1.71 (1.03)	0.247	0.35	0.55	0.223	0.074	0.71	0.471	0.045	1.09
235.99			2.9 (1.43)	2.8 (1.26)	3.9 (1.96)	-0.427	5.90E-07	1.06	-0.051	0.061	0.21	-0.478	0.018	1.21
237.05			1.94 (1.66)	1.08 (0.68)	1.22 (0.88)	0.669	0.0013	1.13	-0.845	0.042	1.79	-0.176	0.25	0.32
242.12			0.62 (0.36)	1.13 (0.76)	0.71 (0.49)	-0.196	0.0099	0.37	0.866	0.00018	1.92	0.67	0.1	1.25
248.19	Hydroxybutyrylcarnitine	HBCt	0.87 (0.49)	1.61 (1.28)	0.91 (0.89)	-0.065	0.46	0.11	0.888	0.001	1.72	0.823	5.00E-04	1.18
250.13			1.22 (0.94)	1.34 (1.02)	1.65 (1.33)	-0.436	0.00067	0.68	0.135	0.59	0.31	-0.3	0.056	0.49
253.01			3 (3.17)	1.62 (1.29)	1.77 (1.64)	0.761	0.006	1.03	-0.889	0.11	1.53	-0.128	0.32	0.19
258.15			5.03 (3.56)	6.38 (5.55)	3.81 (3.44)	0.401	0.0045	0.68	0.343	0.39	0.71	0.744	0.43	1.06
264.31			1.09 (0.64)	0.79 (0.4)	0.68 (0.31)	0.681	2.60E-11	1.63	-0.464	0.0018	1.43	0.216	0.021	0.59
267.3			0.67 (0.49)	0.52 (0.36)	0.68 (0.6)	-0.021	0.45	0.03	-0.366	0.34	0.92	-0.387	0.065	0.6
268.15			14.44 (15.75)	11.91 (13.37)	6.81 (8.73)	1.084	0.00059	1.24	-0.278	0.024	0.45	0.806	0.15	0.87
270.16			2.94 (2.94)	2.27 (2.31)	1.5 (1.77)	0.971	0.00034	1.21	-0.373	0.029	0.66	0.598	0.076	0.72
280.13	Glycerophosphocholine	GPCho	5.9 (3.68)	8.14 (7.62)	4.77 (4.4)	0.307	0.062	0.53	0.464	0.62	0.89	0.771	0.35	1.04
296.11			7.32 (6.71)	12.28 (13.55)	6.25 (7.36)	0.228	0.15	0.29	0.746	0.61	1.09	0.974	0.48	1.06
307.09			5.12 (4.22)	6.19 (4.43)	6.78 (5.66)	-0.405	0.025	0.62	0.274	0.0048	0.63	-0.131	0.41	0.22
322.1			2.47 (2.19)	2.42 (2.08)	1.97 (1.99)	0.326	0.12	0.48	-0.03	0.51	0.07	0.297	0.091	0.42
369.39			1.45 (1.27)	1.75 (1.85)	1.08 (1.19)	0.425	0.00024	0.58	0.271	0.57	0.48	0.696	0.00012	0.83
370.1			1.52 (1.54)	1.54 (1.32)	3.24 (2.97)	-1.092	1.00E-07	1.26	0.019	0.038	0.05	-1.073	0.015	1.31
372.35			0.88 (0.94)	1.29 (1.8)	0.53 (0.74)	0.732	1.60E-09	0.83	0.552	0.0063	0.7	1.283	1.60E-06	1.08
377.99			1.03 (0.64)	1.36 (0.97)	1.7 (1.04)	-0.723	5.20E-09	1.36	0.401	0.0011	0.97	-0.322	0.2	0.66
386.07			1.08 (0.99)	1.24 (1)	2.14 (1.83)	-0.987	1.70E-07	1.24	0.199	0.079	0.4	-0.787	0.0077	1.1
392.11			1.93 (1.87)	1.6 (1.14)	3.84 (3.26)	-0.993	1.90E-08	1.26	-0.271	0.086	0.58	-1.263	0.00036	1.57
399.19			1.33 (0.97)	1.69 (1.19)	1.03 (0.63)	0.369	0.37	0.78	0.346	0.49	0.81	0.714	0.076	1.31
408.08			2.75 (2.91)	2.79 (2.24)	6.03 (5.57)	-1.133	4.90E-11	1.27	0.021	0.021	0.04	-1.112	0.00033	1.34
424.06			1.32 (1.33)	1.56 (1.35)	2.77 (2.69)	-1.069	1.50E-08	1.18	0.241	0.11	0.45	-0.828	0.00062	1.03
427.97			2.22 (1.11)	2.43 (1.08)	3.07 (1.45)	-0.468	1.40E-06	1.19	0.13	0.014	0.48	-0.337	0.033	0.93
428.08			6.96 (7.35)	7.98 (7.8)	14.57 (12.31)	-1.066	2.00E-08	1.31	0.197	0.013	0.34	-0.869	0.0023	1.16
428.42			4.14 (13.42)	4.61 (7.34)	1.57 (4.23)	1.399	2.50E-08	0.58	0.155	0.12	0.12	1.554	8.70E-05	0.97
450.06			2.06 (1.99)	2.06 (1.73)	4.31 (3.41)	-1.065	3.20E-11	1.39	0	0.017	0.01	-1.065	0.0014	1.45
459.3			1.35 (1.14)	0.62 (0.34)	0.54 (0.4)	1.322	1.10E-20	1.91	-1.123	1.80E-08	2.23	0.199	0.02	0.42

466.04			1.59 (1.55)	1.76 (1.54)	3.05 (2.72)	-0.94	1.50E-08	1.16	0.147	0.072	0.28	-0.793	0.001	1.06
472.05			1.77 (1.56)	1.29 (1.05)	3.13 (2.5)	-0.822	2.60E-08	1.16	-0.456	0.46	0.95	-1.279	0.005	1.63
488.02			2.41 (2.36)	2.17 (1.82)	4.77 (3.79)	-0.985	1.40E-09	1.31	-0.151	0.072	0.3	-1.136	3.00E-04	1.51
494.03			0.99 (0.98)	0.79 (0.58)	1.82 (1.48)	-0.878	7.40E-11	1.19	-0.326	0.033	0.66	-1.204	0.01	1.57
504			1.33 (1.29)	1.32 (1.11)	2.5 (2.42)	-0.911	5.50E-09	1.06	-0.011	0.19	0.01	-0.921	0.00018	1.12
508.05			0.91 (1.18)	0.81 (0.77)	1.86 (2.82)	-1.031	6.50E-05	0.77	-0.168	0.48	0.27	-1.199	0.0035	0.92
510.01			1.62 (1.57)	1.33 (1.03)	3.19 (2.41)	-0.978	2.00E-10	1.35	-0.285	0.05	0.6	-1.262	0.00037	1.7
514.28			0.88 (0.66)	0.52 (0.25)	0.45 (0.22)	0.968	7.40E-16	1.81	-0.759	6.70E-06	1.89	0.209	0.047	0.59
518.37	LPC (16:0)	LPC 82	13.7 (12.54)	5.25 (4.88)	4.93 (4.85)	1.475	5.40E-15	1.86	-1.384	7.80E-06	2.26	0.091	0.046	0.13
525.98			1.42 (1.32)	1.35 (1.01)	2.79 (2.37)	-0.974	4.20E-09	1.24	-0.073	0.084	0.17	-1.047	0.00016	1.39
530.03			0.7 (0.75)	0.58 (0.38)	1.23 (1.66)	-0.813	2.90E-05	0.72	-0.271	0.56	0.58	-1.085	0.00077	0.98
531.99			0.78 (0.59)	0.66 (0.34)	1.33 (0.95)	-0.77	1.70E-10	1.21	-0.241	0.12	0.72	-1.011	0.00063	1.6
534.34	LPC (16:0)	LPC 86	11.73 (11.76)	5.22 (4.6)	3.97 (3.71)	1.563	6.30E-13	1.83	-1.168	1.60E-05	1.91	0.395	0.047	0.58
540.29			1.68 (0.84)	1.29 (0.56)	1.29 (0.57)	0.381	3.00E-06	1.09	-0.381	0.011	1.42	0	0.43	0.01
542.12			1 (0.84)	1.82 (1.46)	1.5 (1.02)	-0.585	8.40E-06	1	0.864	0.027	1.6	0.279	0.45	0.49
542.31			1.14 (0.68)	0.76 (0.3)	0.68 (0.28)	0.745	6.10E-17	1.8	-0.585	4.60E-06	1.86	0.16	0.071	0.59
544.39	LPC (18:1)	LPC 90	2.16 (1.35)	1.29 (0.63)	1.36 (0.7)	0.667	6.90E-10	1.51	-0.744	1.00E-04	2.11	-0.076	0.49	0.2
546.26			1.31 (0.65)	1.02 (0.52)	1.36 (0.69)	-0.054	0.5	0.16	-0.361	0.39	1.28	-0.415	0.33	1.04
546.425			2.85 (1.81)	1.59 (0.98)	1.83 (0.93)	0.639	8.80E-10	1.45	-0.842	8.30E-05	2.17	-0.203	0.43	0.48
557.48			0.95 (0.71)	1.1 (0.71)	0.98 (0.79)	-0.045	0.19	0.08	0.212	0.081	0.54	0.167	0.19	0.31
560.36			1.46 (1.19)	0.94 (0.45)	0.71 (0.36)	1.04	1.80E-15	1.78	-0.635	0.00053	1.58	0.405	0.0055	1.05
562.24			1.53 (0.71)	1.21 (0.6)	1.73 (0.75)	-0.177	0.33	0.51	-0.339	0.4	1.28	-0.516	0.014	1.37
562.4			2.38 (1.58)	1.47 (0.84)	1.51 (0.66)	0.656	6.20E-09	1.51	-0.695	1.40E-05	1.85	-0.039	0.51	0.09
564.3			0.94 (0.43)	0.79 (0.28)	1.12 (0.76)	-0.253	0.43	0.53	-0.251	0.15	1.11	-0.504	0.029	1.05
566.37			1.02 (0.58)	0.81 (0.34)	1.16 (0.61)	-0.186	0.28	0.43	-0.333	0.48	1.2	-0.518	0.036	1.25
568.3			0.97 (0.3)	0.92 (0.29)	1 (0.36)	-0.044	0.46	0.17	-0.076	0.55	0.47	-0.12	0.14	0.49
578.26			0.86 (0.37)	0.79 (0.33)	0.93 (0.42)	-0.113	0.41	0.35	-0.122	0.37	0.48	-0.235	0.0096	0.68
580.07			1.08 (1)	2.23 (1.9)	1.6 (1.22)	-0.567	6.40E-06	0.86	1.046	0.0043	1.73	0.479	0.49	0.77
582.35			0.87 (0.44)	0.75 (0.3)	0.96 (0.41)	-0.142	0.26	0.39	-0.214	0.35	0.88	-0.356	0.0027	1.07
586.08			0.98 (0.79)	1.55 (1.13)	1.69 (1.04)	-0.786	8.40E-09	1.38	0.661	0.0011	1.4	-0.125	0.55	0.25
586.29			0.77 (0.29)	0.74 (0.26)	0.85 (0.37)	-0.143	0.2	0.44	-0.057	0.45	0.29	-0.2	0.52	0.64
590.29			1.27 (0.46)	1.06 (0.39)	1.53 (0.54)	-0.269	0.001	0.97	-0.261	0.46	1.25	-0.529	2.00E-04	1.71
602.06			1.53 (1.51)	3.05 (2.45)	2.68 (1.88)	-0.809	1.00E-07	1.23	0.995	0.0012	1.73	0.187	0.52	0.33
605.6			1.17 (0.51)	1.09 (0.47)	1.01 (0.5)	0.212	0.0048	0.61	-0.102	0.15	0.42	0.11	0.18	0.31
616.23			1.49 (2.05)	1.02 (1.12)	1.24 (2.17)	0.265	0.0068	0.24	-0.547	0.31	0.78	-0.282	0.37	0.24
616.42			0.66 (0.27)	0.64 (0.27)	0.88 (0.64)	-0.415	0.00083	0.76	-0.044	0.36	0.19	-0.459	0.084	0.86
624.04			1.03 (0.65)	1.53 (0.91)	1.53 (0.89)	-0.571	1.60E-07	1.16	0.571	0.00072	1.5	0	0.47	0
627.95			0.66 (0.42)	0.57 (0.23)	0.97 (0.95)	-0.556	5.50E-07	0.76	-0.212	0.57	0.67	-0.767	6.40E-05	1.03
635.2			0.88 (0.44)	0.62 (0.32)	1.01 (0.5)	-0.199	0.039	0.53	-0.505	0.12	1.67	-0.704	0.001	1.58
651.18			0.89 (0.46)	0.79 (0.45)	1.12 (0.69)	-0.332	0.0012	0.71	-0.172	0.58	0.6	-0.504	0.00014	1.05

664.18			1.33 (1.68)	3.49 (3.56)	2.3 (1.95)	-0.79	4.30E-06	0.99	1.392	0.00033	1.76	0.602	0.5	0.81
672.48			2.06 (0.89)	2.32 (1.2)	1.75 (0.7)	0.235	0.0011	0.78	0.171	0.072	0.59	0.407	1.90E-05	1.09
688.46			2.11 (0.96)	2.59 (1.11)	1.79 (0.67)	0.237	0.0013	0.79	0.296	0.016	1.13	0.533	8.30E-07	1.57
690.07			1.13 (0.77)	1.22 (0.84)	0.84 (0.46)	0.428	0.039	0.98	0.111	0.53	0.27	0.538	0.013	1.09
697.59			1.37 (0.73)	1 (0.37)	1.25 (0.49)	0.132	0.073	0.42	-0.454	0.24	1.68	-0.322	0.25	1.05
700.51			0.97 (0.37)	1.01 (0.43)	0.99 (0.32)	-0.029	0.44	0.09	0.058	0.13	0.27	0.029	0.038	0.14
706.04			1.05 (0.71)	1.25 (0.82)	0.79 (0.44)	0.41	0.03	0.93	0.252	0.53	0.64	0.662	0.06	1.31
706.61			1.11 (0.55)	1.38 (0.92)	1.14 (0.47)	-0.038	0.53	0.11	0.314	0.22	0.84	0.276	0.028	0.64
713.56			1.01 (0.45)	0.84 (0.27)	0.93 (0.29)	0.119	0.041	0.45	-0.266	0.21	1.22	-0.147	0.26	0.62
716.5			1 (0.37)	1.12 (0.41)	1.01 (0.32)	-0.014	0.43	0.1	0.163	0.016	0.78	0.149	0.0092	0.55
723.61			1.48 (0.54)	1.28 (0.41)	1.48 (0.49)	0	0.43	0.01	-0.209	0.18	1.11	-0.209	0.24	0.83
725.63	SM (34:1;2)	SM 125	22.7 (10.97)	16.71 (9.83)	23.52 (9.95)	-0.051	0.53	0.16	-0.442	0.13	1.44	-0.493	0.13	1.26
728.59			2.06 (0.92)	2.42 (1.37)	2.18 (0.86)	-0.082	0.41	0.28	0.232	0.077	0.76	0.151	0.022	0.41
731.67			1.29 (0.5)	1.28 (0.57)	1.14 (0.44)	0.178	0.0023	0.64	-0.011	0.53	0.03	0.167	0.0069	0.55
732.62			2.17 (0.92)	2.46 (1.54)	1.7 (0.69)	0.352	2.10E-05	1.13	0.181	0.57	0.57	0.533	1.70E-05	1.21
734.64			5.68 (3.36)	5.59 (3.57)	6.57 (3.58)	-0.21	0.38	0.5	-0.023	0.59	0.07	-0.233	0.54	0.53
740.58			1.3 (0.64)	1.21 (0.54)	0.79 (0.27)	0.719	3.10E-19	2.03	-0.104	0.18	0.37	0.615	2.70E-09	1.78
741.6			14.16 (7.19)	11.98 (5.27)	14.4 (5.99)	-0.024	0.5	0.07	-0.241	0.098	0.91	-0.265	0.033	0.81
744.58			1.95 (0.88)	2.51 (1.4)	1.9 (0.71)	0.037	0.44	0.12	0.364	0.095	1.15	0.402	0.02	1.05
753.66			2.82 (1.42)	2.74 (1.5)	3.56 (2)	-0.336	0.031	0.78	-0.042	0.54	0.15	-0.378	0.49	0.86
754.525			2.76 (1.29)	3.61 (2.26)	2.41 (1)	0.196	0.021	0.61	0.387	0.035	1.1	0.583	9.40E-06	1.3
756.62	PC (16:0_16:0)	PC 135	10.48 (6.45)	10.04 (5.99)	13.2 (5.91)	-0.333	0.04	0.86	-0.062	0.45	0.19	-0.395	0.41	0.99
758.64			10.13 (5.8)	10.27 (7.68)	8.29 (4.02)	0.289	9.60E-05	0.76	0.02	0.5	0.05	0.309	0.0081	0.64
760.66	PC (16:0_18:1)	PC 137	21.89 (15.12)	22.35 (13.42)	10.16 (6.96)	1.107	1.00E-17	1.95	0.03	0.47	0.08	1.137	3.50E-09	1.96
769.64			2.47 (1.06)	2.81 (1.23)	2.65 (1.17)	-0.101	0.48	0.31	0.186	0.33	0.75	0.085	0.37	0.27
770.49			2.47 (1.17)	3.47 (1.81)	2.08 (0.87)	0.248	0.0051	0.78	0.49	0.026	1.54	0.738	9.50E-07	1.75
772.6	PC (32:0)	PC 140	8.03 (4.89)	9.26 (6.05)	9.92 (4.2)	-0.305	0.031	0.82	0.206	0.36	0.56	-0.099	0.37	0.25
778.56			2.17 (1.48)	2.12 (1.08)	0.87 (0.48)	1.319	1.40E-30	2.27	-0.034	0.096	0.1	1.285	4.90E-14	2.37
780.63	PC (16:0_18:2)	PC 142	16.57 (8.79)	16.02 (9.44)	12.52 (5.81)	0.404	1.20E-06	1.1	-0.049	0.5	0.15	0.356	0.00056	0.86
782.65	PC (16:0_18:1)	PC 143	45.62 (25.39)	46.11 (24.92)	23.94 (12.64)	0.93	1.70E-20	2.06	0.015	0.52	0.05	0.946	1.80E-08	1.94
790.61			2.12 (0.86)	2.04 (0.9)	1.5 (0.62)	0.499	4.70E-13	1.6	-0.055	0.47	0.22	0.444	3.40E-06	1.32
796.61			13.98 (8.84)	15.65 (9.34)	10.5 (5.65)	0.413	2.30E-05	0.97	0.163	0.48	0.47	0.576	0.0032	1.25
798.62	PC (34:1)	PC 146	34.31 (22.56)	39.98 (20.45)	15.17 (9.41)	1.177	2.60E-21	2.13	0.221	0.5	0.68	1.398	5.00E-12	2.43
804.63	PC (36:4)	PC 147	7.35 (3.85)	6.51 (3.6)	9 (4.4)	-0.292	0.048	0.76	-0.175	0.53	0.58	-0.467	0.14	1.14
806.65			7.68 (3.9)	6.71 (3.2)	6.08 (2.53)	0.337	2.20E-06	0.99	-0.195	0.37	0.7	0.142	0.0084	0.43
808.67	PC (36:2)	PC 149	13.59 (6.65)	11.61 (6.56)	9.46 (4.45)	0.523	8.70E-11	1.44	-0.227	0.34	0.76	0.295	0.00015	0.74
810.68	PC (36:1)	PC 150	10.32 (4.68)	10.42 (6.16)	7.85 (3.58)	0.395	2.20E-08	1.17	0.014	0.51	0.04	0.409	5.90E-05	0.98
812.535			2.38 (0.87)	2.28 (0.82)	2.04 (0.78)	0.222	0.00019	0.82	-0.062	0.5	0.32	0.16	0.0068	0.58
812.775			2.81 (1.28)	2.69 (1.2)	2.23 (0.93)	0.334	2.10E-06	1.04	-0.063	0.52	0.24	0.271	0.0022	0.83
820.61	PC (36:4)	PC 153	5.5 (2.97)	5.86 (3.26)	6.97 (3.17)	-0.342	0.0069	0.91	0.091	0.28	0.29	-0.25	0.034	0.66

822.62	PC (36:3)	PC 154	5.71 (2.74)	5.79 (2.57)	4.6 (2.16)	0.312	1.40E-05	0.9	0.02	0.42	0.08	0.332	0.065	0.95
824.64	PC (36:2)	PC 155	9.94 (5.34)	9.62 (4.83)	6.68 (3.65)	0.573	1.60E-11	1.41	-0.047	0.19	0.16	0.526	0.0011	1.27
826.66	PC (36:1)	PC 156	6.01 (3.12)	7.04 (3.63)	3.54 (1.83)	0.764	9.30E-19	1.85	0.228	0.43	0.76	0.992	1.70E-08	2.06
830.65			2.2 (1.01)	2.06 (0.81)	2.06 (0.83)	0.095	0.094	0.3	-0.095	0.57	0.39	0	0.16	0.01
832.67	PC (38:4)	PC 158	5.53 (3.63)	4.76 (2.96)	6.67 (3.22)	-0.27	0.15	0.66	-0.216	0.5	0.61	-0.487	0.17	1.14
834.68			3.36 (1.83)	2.85 (1.39)	3.18 (1.41)	0.079	0.094	0.23	-0.237	0.5	0.83	-0.158	0.54	0.46
835.75			3.96 (2.09)	3.54 (2.1)	4.72 (2.17)	-0.253	0.046	0.69	-0.162	0.5	0.51	-0.415	0.099	1.03
837.77			3.89 (1.94)	3.51 (2.08)	4.01 (1.96)	-0.044	0.47	0.12	-0.148	0.44	0.48	-0.192	0.55	0.48
846.63			1.64 (0.74)	1.74 (0.67)	1.61 (0.64)	0.027	0.31	0.11	0.085	0.48	0.35	0.112	0.37	0.39
848.65	PC (38:4)	PC 163	4.17 (2.67)	4.26 (2.69)	5.11 (2.51)	-0.293	0.054	0.71	0.031	0.39	0.09	-0.262	0.043	0.63
850.66	PC (38:3)	PC 164	2.59 (1.24)	2.44 (0.98)	2.58 (1.28)	0.006	0.35	0.02	-0.086	0.45	0.36	-0.08	0.17	0.24
851.73			2.54 (1.26)	2.56 (1.24)	2.92 (1.19)	-0.201	0.067	0.61	0.011	0.55	0.05	-0.19	0.022	0.57
853.75			2.6 (1.48)	2.5 (1.15)	2.54 (1.15)	0.034	0.28	0.1	-0.057	0.22	0.21	-0.023	0.31	0.07
926.65			0.89 (0.58)	0.88 (0.49)	1.53 (1.37)	-0.782	0.00031	1.06	-0.016	0.27	0	-0.798	0.0058	1.12
